# Supplementary material for: Attenuation of SARS‐CoV‐2 replication and associated inflammation by concomitant targeting of viral and host cap 2'‐O‐ribose methyltransferases
Source: EMBO J. 2022 Jul 25;41(17):e111608. doi: 10.15252/embj.2022111608 (PMC9350232; doi:10.15252/embj.2022111608)
Supplement: Supplementary file 1 — Appendix [file EMBJ-41-e111608-s002.pdf]

Appendix

Table of Contents

Appendix Figure S1 2

Legend 3

Appendix Figure S1

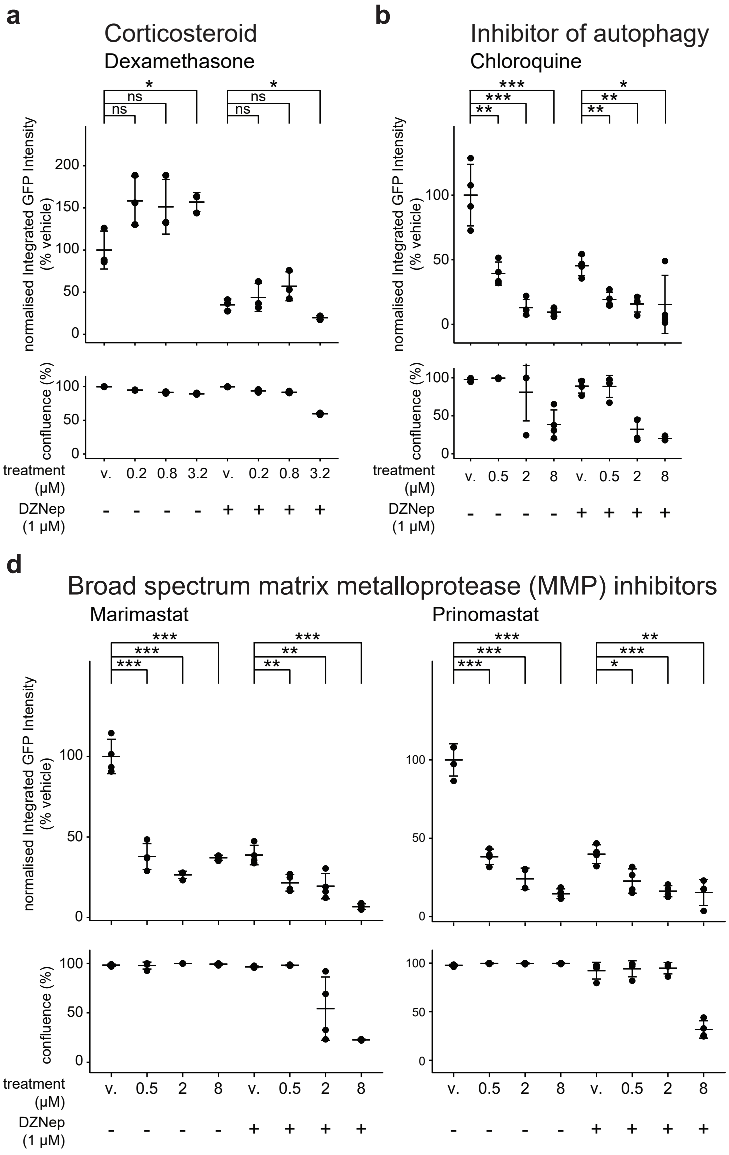

Appendix Figure S1 | (a-d) A549-ACE2 cells were pretreated for 6 h with the indicated concentrations of **(a)** dexamethasone, **(b)** chloroquine, **(c)** Ipatasertib or **(d)** Marimastat (left) and Prinomastat (right), and infected with SARS-CoV-2-GFP at MOI 3. Normalized integrated GFP intensity and confluence at 72h post infection are depicted as measures of virus replication and cell growth, respectively. Plots depict mean +/- sd of **(a)** 3 or **(b, c, d)** 4 independently infected wells. Statistics were calculated using Student's two-sided t-test between indicated treatment concentrations and vehicle controls. ns $p>0.05$, * $p<0.05$, ** $p<0.01$, *** $p<0.001$.
