# Supplementary material for: Attenuation of SARS‐CoV‐2 replication and associated inflammation by concomitant targeting of viral and host cap 2'‐O‐ribose methyltransferases
Source: EMBO J. 2022 Jul 25;41(17):e111608. doi: 10.15252/embj.2022111608 (PMC9350232; doi:10.15252/embj.2022111608)
Supplement: Supplementary file 2 — Expanded View Figures PDF [file EMBJ-41-e111608-s003.pdf]

Expanded View Figures

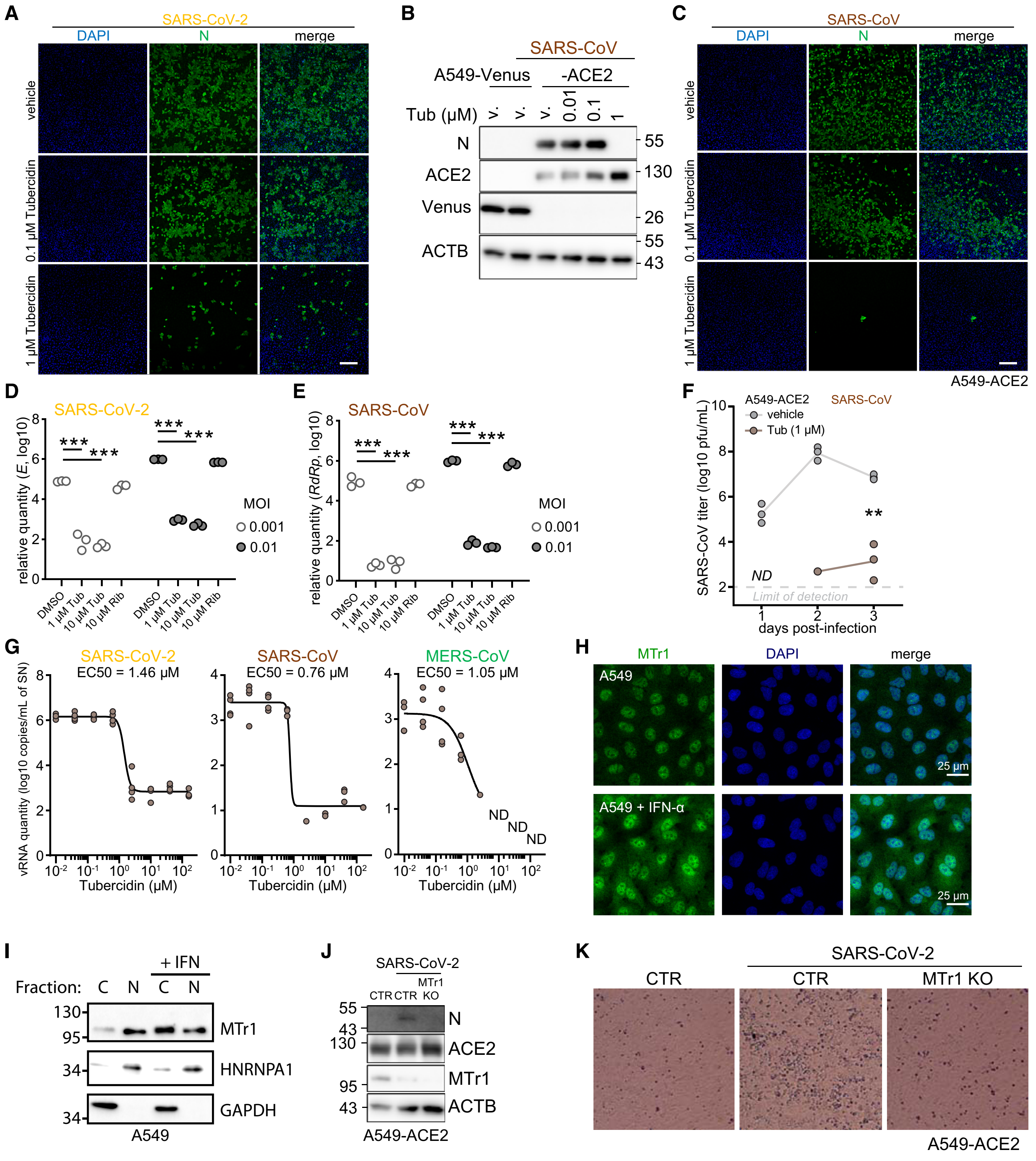

Figure EV1.

 © 2022 The Authors

Figure EV1. ***In silico*** **screening identified NSP16 inhibitors with potent anti-SARS-CoV-2 activity.**

A Representative images from three independent immunofluorescent analyses of A549-ACE2 cells, pretreated for 3 h with indicated concentrations of Tubercidin or vehicle (DMSO) and infected with SARS-CoV-2 at MOI 0.5 at 24 h post-infection. Immunostaining against the viral N protein is shown in green alongside DAPI staining in the blue channel. Scale bar: 250 μm.

B A549-ACE2 or control cell line A549-Venus were pretreated for 3 h with Tubercidin or vehicle (DMSO) and infected with SARS-CoV at MOI 0.1. After 24 h, abundance of SARS-CoV nucleoprotein (N), ACE2, Venus and β-actin (ACTB, loading control) was visualized using Western blotting. Presented data is representative of three independent experiments.

C Similar to (A), but the cells were infected with SARS-CoV. Scale bar: 250 μm. Representative images from three independent immunofluorescent analyses.

D, E A549-ACE2 cells pretreated for 3 h with vehicle (DMSO), Tubercidin or Ribavirin and infected with SARS-CoV-2 (D) or SARS-CoV (E) at the indicated MOIs. At 24 h post infection, relative expression of SARS-CoV-2 *E* was quantified by RT-qPCR. Statistics were calculated using Student's two-sided t-test between the three independent infections of the indicated conditions. The measurements are representative of three independent experiments. *** $P < 0.001$.

F A549-ACE2 cells were pretreated for 3 h with Tubercidin or vehicle (DMSO) and infected with SARS-CoV at MOI 0.01. At 1h post-infection, medium change was performed. At the indicated days post infection, infectious viral progeny was quantified in the supernatant of $n = 3$ independently infected wells by plaque assay on Vero cells. The presented data is representative of two independent experiments. *ND*, not detected. Statistics were calculated using Student's two-sided t-test. ** $P < 0.01$.

G Vero E6 cells expressing TMPRSS2 were pretreated for 3 h with the indicated concentration of Tubercidin and infected with SARS-CoV-2 (left), SARS-CoV (middle) or MERS-CoV (right) at MOI 0.1. At 1h post-infection, medium change was performed. At 24 hours post-infection, viral RNA in the supernatant of $n = 4$ independently infected wells was isolated and quantified using RT-qPCR as a measure of virus replication. SARS-CoV-2 *E*, SARS-CoV *N*, and MERS-CoV *N* coding regions were targeted for quantification.

H A549 cells were treated with vehicle or IFN-α at 500 U/ml overnight. The cells were fixed with 4% PFA and stained with anti-MTr1 antibody and DAPI.

I A549 cells were treated with IFN-β at 1,000 U/ml overnight followed by nucleo-cytoplasmic fractionation. Quantification of MTr1, cytoplasmic marker GAPDH and nuclear marker hnRNP A1 was performed by Western blotting. Presented data is representative of three independent repeats.

J A549-ACE2 CTR or MTr1 KO cell lines were infected with SARS-CoV-2 at MOI 0.1. After 24 h, abundances of viral nucleoprotein (N), ACE2, MTr1 and β-actin (ACTB, loading control) were visualized using Western blotting. Presented data is representative of three independent experiments.

K Representative bright-field images of A549-ACE2 CTR and MTr1 KO cells infected with SARS-CoV-2 at MOI 0.01 at 72 h post-infection. Images are representative of two independent experiments.

Source data are available online for this figure.

© 2022 The Authors

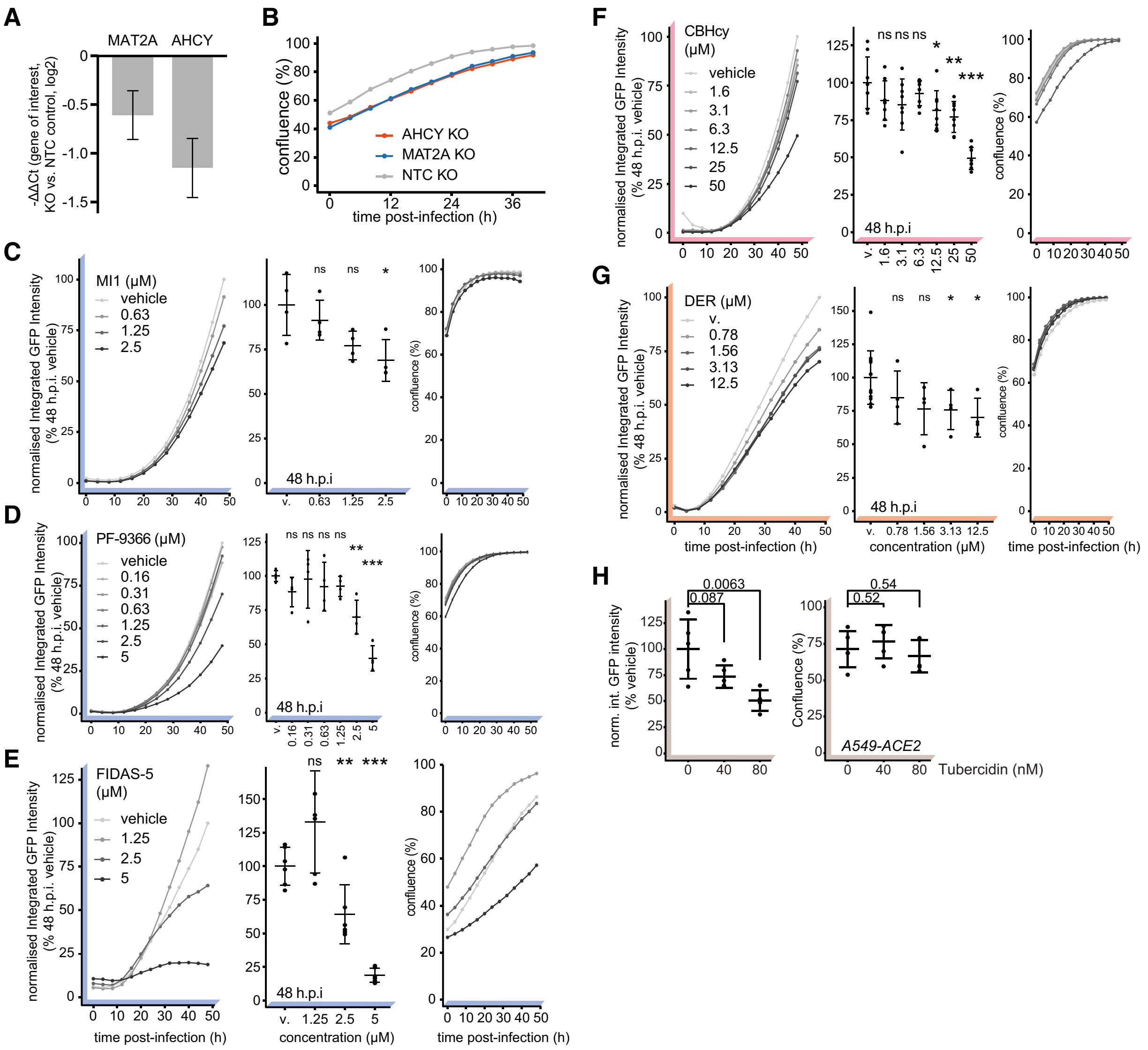

Figure EV2. SAM-cycle enzymes are key host factors facilitating SARS-CoV-2 proliferation.

A Negative ΔΔCt values depicting reduction in expression of *MAT2A* and *AHCY* mRNA in relevant KOs versus NTC (targets normalized to *RPLP0*). Data depicts mean ± propagated SD of four technical (RT-qPCR) replicates.

B A549-ACE2 cells with CRISPR/Cas9 mediated genetic ablation of *AHCY* or *MAT2A* (or using non-targeting control, NTC) were infected with SARS-CoV-2-GFP at MOI 3 and confluence plotted over time as measure of cell growth. Data depicts means of three independently infected wells.

C–H A549-ACE2 cells were pretreated for 6 h with indicated concentrations of (C) MI1, (D) PF-9366, (E) FIDAS-5, (F) CBHcy, (G) DER or (H) Tubercidin and infected with SARS-CoV-2-GFP at MOI 3. Normalized integrated GFP intensity and confluence are depicted as measures of virus replication and cell growth, respectively. Plots depict mean ± SD of (C–E) 4, (F) 6, (G) 4 (12 vehicle) or (H) 5 (24 h.p.i.) independently infected wells. Statistics were calculated using Student's two-sided *t*-test between indicated treatment concentrations and vehicle controls (MI1, PF-9366, FIDAS-5, Tubercidin – DMSO; CBHcy, DER – PBS). ns $P > 0.05$, * $P < 0.05$, ** $P < 0.01$, *** $P < 0.001$.

Source data are available online for this figure.

 © 2022 The Authors

Figure EV3. SAM-cycle inhibitor DZNep retains antiviral activity against SARS-CoV-2 in STAT1-deficient cells.

A–C Vero E6 cells were pretreated for 6 h with indicated concentrations of (A) DZNep, (B) FIDAS-5 and (C) CBHcy, and infected with SARS-CoV-2 at MOI 0.01. 48 h post-infection, produced infectious progeny was titrated on Vero E6 cells. Error bars correspond to mean ± SD of $n = 3$ independently infected wells.

D Western blot showing abundances of SARS-CoV-2 nucleoprotein (N) and host β-actin (ACTB, loading control) upon 6 h pretreatment of A549-ACE2s with indicated concentrations of DZNep or vehicle (v., PBS) and infection with SARS-CoV-2 at MOI 3 for 24 h.

E Similar to (D), with abundances of SARS-CoV and SARS-CoV-2 N depicted alongside the percentage of N signal normalized to the vehicle-treated sample (relative to loading control).

F A549-ACE2 cells were pretreated for 6 h with indicated concentrations of DZNep (right), IFN-α (middle) and infected with SARS-CoV-2-GFP at MOI 3. Alternatively, the virus inoculum was incubated with indicated dilutions of antisera for 1 h at 37°C before use (left). Logistic curves, fitted to normalized integrated GFP intensity are depicted as measures of virus replication alongside maximum derivative at the inflection points (dashed lines) and respective linear regression intercepts with zero (diamonds). Depicted findings are based on 4 (DZNep), 2 (IFN-α) and 12 (antisera) independently infected wells.

G A549-ACE2 cells were treated with DZNep (2 μM) or vehicle (PBS) at 4 h prior to, at the time of, or 4 h post-infection with SARS-CoV-2-GFP at MOI 3. Normalized integrated GFP intensity is depicted as a measure of reporter virus replication. Plot depicts individual measurements (offset for clarity) alongside mean ± SD of 6 (DZNep) or 4 (vehicle) independently infected wells.

H STAT1 KO or NTC A549-ACE2 cells were pretreated for 6 h with the indicated concentration of interferon α (IFN-α), DZNep or PBS (vehicle), and infected with SARS-CoV-2-GFP at MOI 3. Normalized GFP area is plotted over time as a measure of reporter virus growth. Data shows mean of three independently infected wells and is representative of three independent repeats.

I A549-ACE2 cells were pretreated for 6 h with indicated concentrations of Tazemetostat and infected with SARS-CoV-2-GFP at MOI 3. Normalized integrated GFP intensity is plotted over time (left) and at 48 h post-infection (right) as a measure of reporter virus growth. Means of four independently infected wells (left) and means ± SD (right) are depicted. Statistics were calculated using Student's two-sided *t*-test between indicated treatment concentrations and vehicle control (v., DMSO). * $P < 0.05$, ** $P < 0.01$, *** $P < 0.001$.

Source data are available online for this figure.

© 2022 The Authors

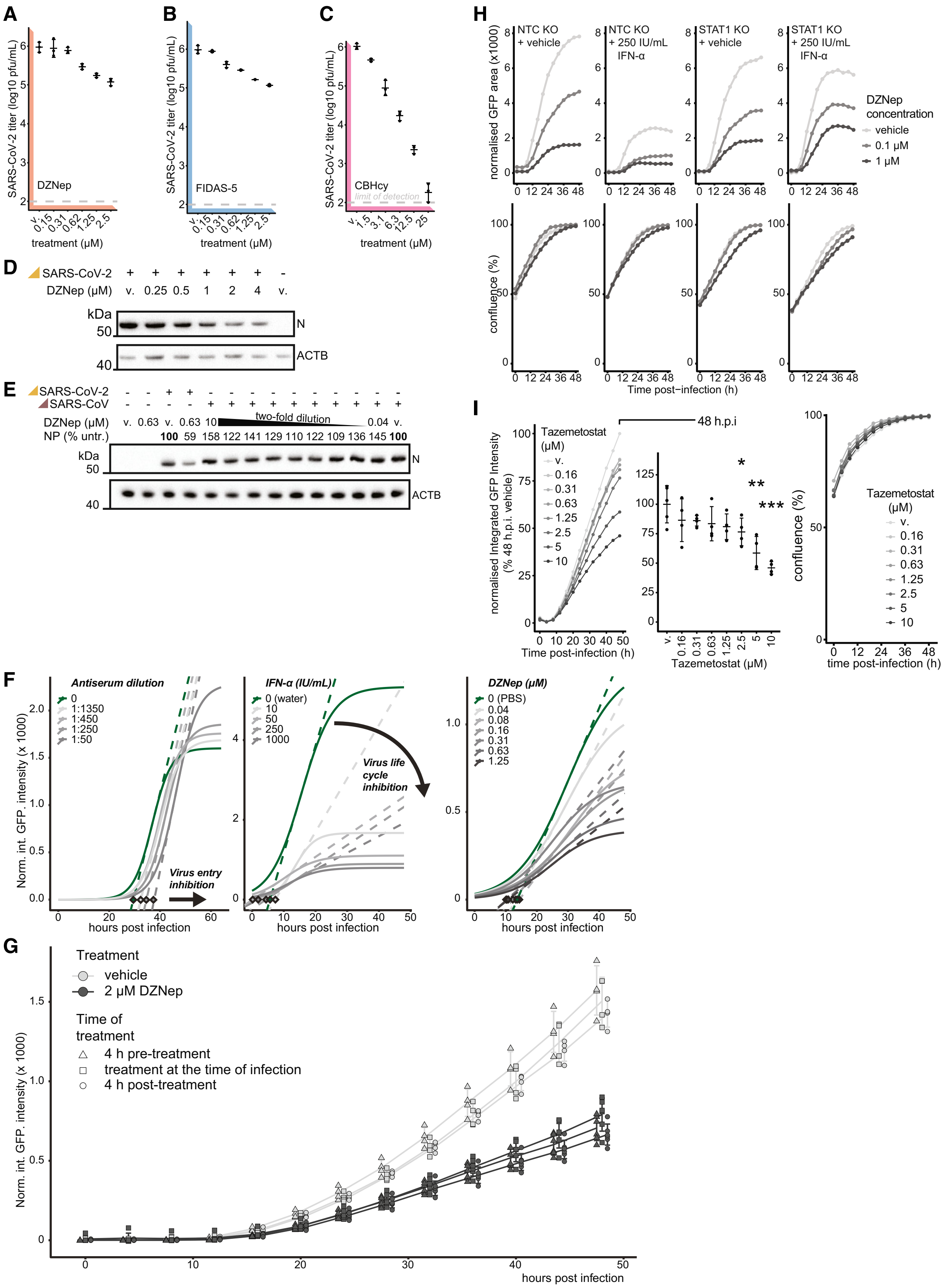

Figure EV3.

© 2022 The Authors

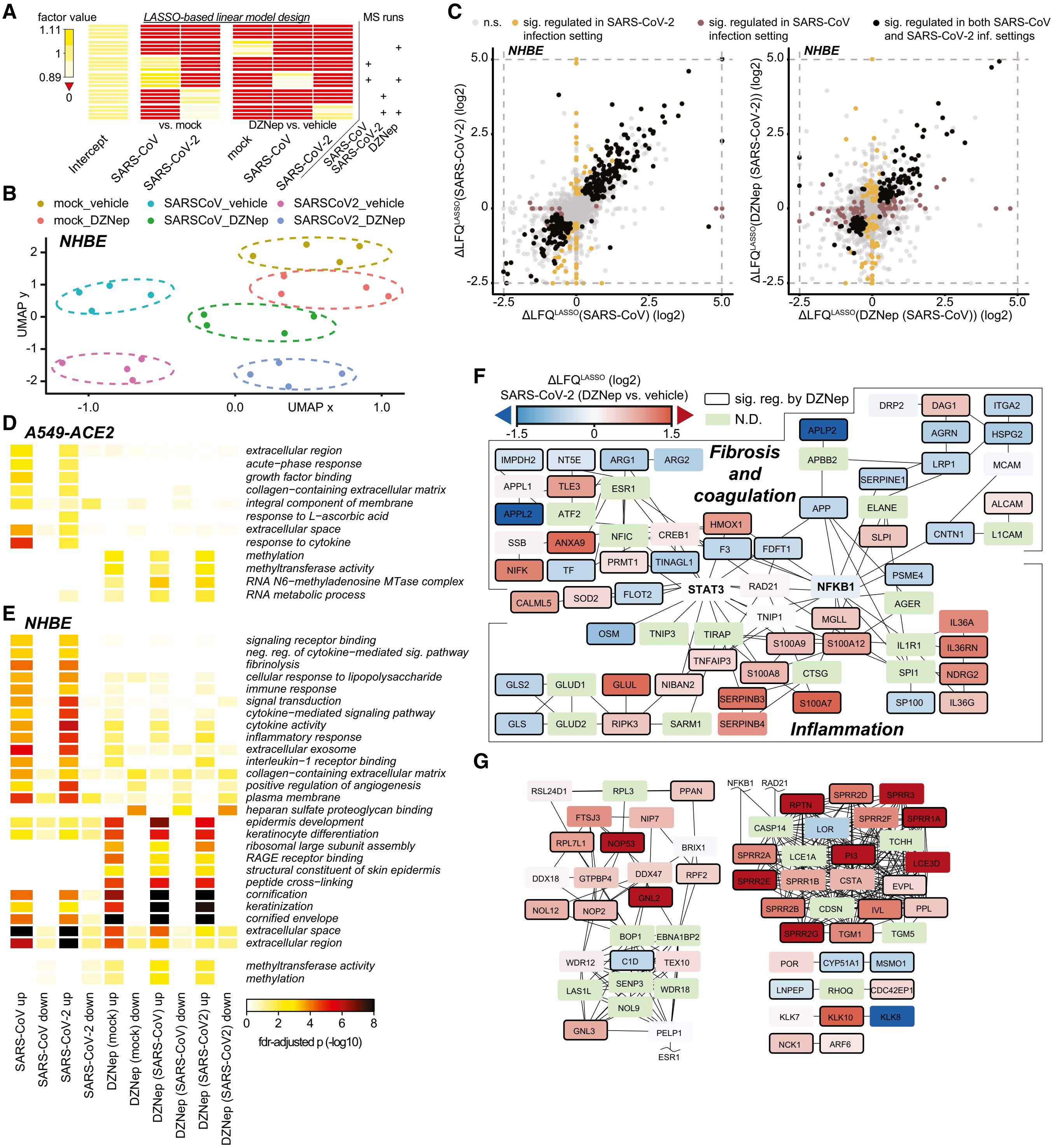
A
factor value
LASSO-based linear model design
MS runs
vs. mock
DZNep vs. vehicle
Intercept
SARS-CoV
SARS-CoV-2
mock
DZNep
B
mock_vehicle
mock_DZNep
SARSCoV_vehicle
SARSCoV_DZNep
SARSCoV2_vehicle
SARSCoV2_DZNep
NHBE
UMAP y
UMAP x
C
n.s.
sig. regulated in SARS-CoV-2 infection setting
sig. regulated in SARS-CoV infection setting
sig. regulated in both SARS-CoV and SARS-CoV-2 inf. settings
D
A549-ACE2
extracellular region
acute−phase response
growth factor binding
collagen−containing extracellular matrix
integral component of membrane
response to L−ascorbic acid
extracellular space
response to cytokine
methylation
methyltransferase activity
RNA N6−methyladenosine MTase complex
RNA metabolic process
E
signaling receptor binding
neg. reg. of cytokine−mediated sig. pathway
fibrinolysis
cellular response to lipopolysaccharide
immune response
signal transduction
cytokine−mediated signaling pathway
cytokine activity
inflammatory response
extracellular exosome
interleukin−1 receptor binding
positive regulation of angiogenesis
plasma membrane
heparan sulfate proteoglycan binding
epidermis development
keratinocyte differentiation
ribosomal large subunit assembly
RAGE receptor binding
structural constituent of skin epidermis
peptide cross−linking
cornification
keratinization
cornified envelope
fdr-adjusted p (-log10)
SARS-CoV up
SARS-CoV down
SARS-CoV-2 up
SARS-CoV-2 down
DZNep (mock) up
DZNep (mock) down
DZNep (SARS-CoV) up
DZNep (SARS-CoV) down
DZNep (SARS-CoV2) up
DZNep (SARS-CoV2) down
F
SARS-CoV-2 (DZNep vs. vehicle)
sig. reg. by DZNep
N.D.
Fibrosis and coagulation
Inflammation
STAT3
NFKB1
G

Figure EV4.

Figure EV4. DZNep treatment modulates tissue and immune processes.

A Experimental design matrix used for LASSO-based linear model depicting relationship between MS runs (samples) and coefficients in the model. Values of factors, used in the modeling approach and shown in the heatmap, were calculated as described in Materials and Methods.

B UMAP dimensionality reduction, applied to normalized LFQ protein abundances of DZNep- or vehicle-treated and SARS-CoV, SARS-CoV-2 or mock-infected NHBEs.

C Scatterplots depicting the relationship between SARS-CoV-2 and SARS-CoV induced protein changes relative to mock-infection (left) and DZNep induced protein changes in the context of SARS-CoV-2 and SARS-CoV infection (right) of NHBEs. LASSO-based $\log_2$ fold-changes are depicted. n.s., not significant.

D, E Heatmap depicting GO-terms, enriched in at least two comparisons (columns) of A549 (D) or NHBE (E) proteome analyses at fdr-adjusted *P*-values < 0.025 and < 0.001, respectively. For NHBEs, the thresholds do not apply to the methylation-related (bottom two) terms. Statistics were calculated using Fisher exact test with FDR-based *P*-value adjustment.

F, G Proteins, differentially expressed upon DZNep treatment of NHBEs in the contexts of SARS-CoV and SARS-CoV-2 infections were used for network diffusion analysis in order to identify genes functionally interacting with them. (F) The graph shows a cluster of genes found to be significantly enriched in this analysis and that was related to fibrosis and coagulation, and inflammation. (G) Sections of significant genes from network diffusion analysis of DZNep-regulated proteins outside the fibrosis and inflammation related cluster (F). The networks are overlaid with LASSO-based $\log_2$ fold change between SARS-CoV-2 infected DZNep- and vehicle-treated NHBEs. N.D., not detected.

Figure EV5. DZNep treatment does not lead to virus adaptation, exhibits synergism with Remdesivir and IFN-α and is antiviral *in vivo*.

A–E Related to the virus adaptation experiment (Fig 5A, left). (A) Titer of viral progeny after each passage of SARS-CoV-2 on Vero E6 cells (MOI 0.01, 48 h) in the presence of the indicated treatments. (B) Exemplar read coverage profiles (left) and mean coverage of SARS-CoV-2 genome as annotated. (C) Number of synonymous and non-synonymous mutations, arising at or above indicated frequencies in depicted sequenced viral genomes relative to the Wuhan-Hu-1 reference genome sequence. (D) Phylogenetic representation of sequenced viral genomes. (E) Heatmap representing a subset of variation landscape during the virus adaptation. Selected variations are depicted alongside corresponding SARS-CoV amino acids at indicated positions and variation frequencies. Full table of detected variations and their respective frequencies is available in the Dataset EV5.

F Ratio of indicated pairs of viral isolates in 1:1 inoculums and 24 and 48 h post infection of Vero E6 cells undergoing treatments as annotated. Six (DMSO P10/FIDAS-5 P10) and 4 (DMSO P10/P0) individual variations were used for ratio calculation as further depicted in the panel (G), and are shown alongside means ± SD.

G Mutations, detected in sequenced viral genomes in virus competition assay (full list available in Dataset EV6) that were used for calculation of ratios between isolates (Figs 5B and EV5F).

H A549-ACE2 cells were pretreated with indicated concentrations of IFN-α and DZNep and infected with SARS-CoV-2-GFP at MOI 1. Cell confluence is depicted as a measure of cell growth at 24 h post-infection. Means of six independently infected wells are shown.

I A549-nRFP-ACE2 cells, i.e. A549-ACE2 cells expressing nuclear red fluorescent protein, were pretreated with indicated concentrations of Remdesivir and DZNep and infected with SARS-CoV-2-GFP at MOI 1. Number of red objects (RFP-positive cell nuclei) as a measure of cell growth at 24 h post-infection is depicted. Means of five independently infected wells are shown and the data is representative of three independent repeats.

J C57BL/6 mice were infected with SARS-CoV-2 beta variant (250 pfu, intranasal) and treated at D0 and D1 with DZNep (10 μg, intranasal). Forty-eight hours post infection, lungs of infected mice were isolated. Abundance of viral transcripts encoding SARS-CoV-2 membrane protein (*M*) and envelope protein (*E*) were quantified in the lung samples by RT-qPCR. The graph shows negative ΔCt values, as normalized to *18S rRNA*, and respective mean ± SD ($n = 8$ animals per condition). The presented data was pooled from two independent experiments. Statistics were calculated using Student's two-sided *t*-test as indicated.

 © 2022 The Authors

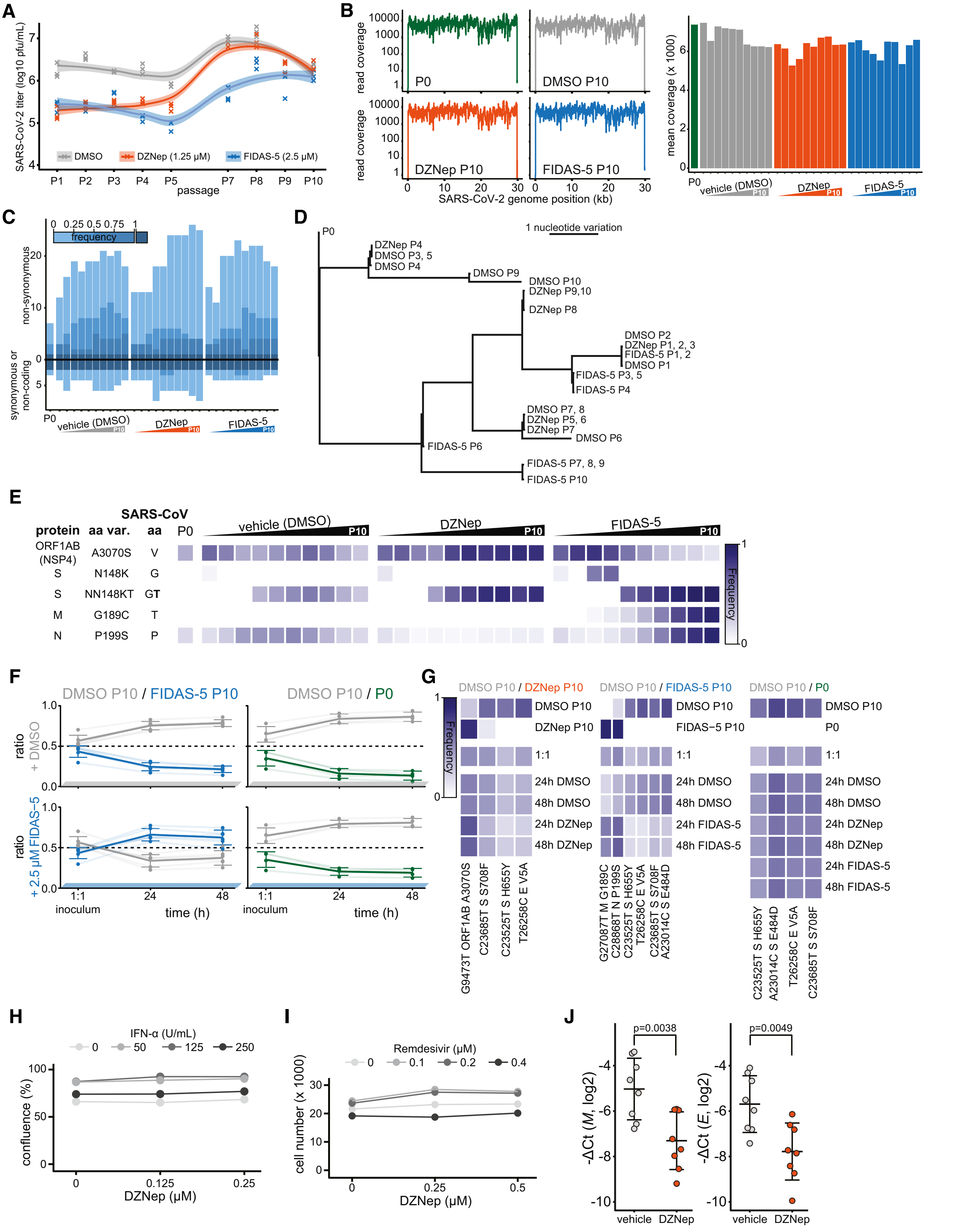
A
SARS-CoV-2 titer (log10 pfu/mL)
DMSO
DZNep (1.25 µM)
FIDAS-5 (2.5 µM)
passage
B
read coverage
P0
DMSO P10
DZNep P10
FIDAS-5 P10
SARS-CoV-2 genome position (kb)
mean coverage (x 1000)
vehicle (DMSO)
DZNep
FIDAS-5
C
frequency
non-synonymous
synonymous or non-coding
D
1 nucleotide variation
E
protein
aa var.
SARS-CoV aa
ORF1AB (NSP4)
A3070S
N148K
NN148KT
G189C
P199S
Frequency
F
DMSO P10 / FIDAS-5 P10
DMSO P10 / P0
ratio + DMSO
ratio + 2.5 µM FIDAS-5
inoculum
time (h)
G
DMSO P10 / DZNep P10
DMSO P10 / FIDAS-5 P10
DMSO P10 / P0
24h DMSO
48h DMSO
24h DZNep
48h DZNep
24h FIDAS-5
48h FIDAS-5
H
IFN-α (U/mL)
confluence (%)
DZNep (µM)
I
Remdesivir (µM)
cell number (x 1000)
DZNep (µM)
J
p=0.0038
p=0.0049
-ΔCt (M, log2)
-ΔCt (E, log2)
vehicle
DZNep

Figure EV5.
