# Supplementary figures and images for: Attenuation of SARS‐CoV‐2 replication and associated inflammation by concomitant targeting of viral and host cap 2'‐O‐ribose methyltransferases
Source: EMBO J. 2022 Jul 25;41(17):e111608. doi: 10.15252/embj.2022111608 (PMC9350232; doi:10.15252/embj.2022111608)

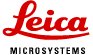

Supplement: Supplementary file 9 — Source Data for Expanded View [file EMBJ-41-e111608-s010.zip › SourceData/Figure_EV1/FEV1a/SARS2 0.1uM Tub/MetaData/LeicaLogo.jpg]

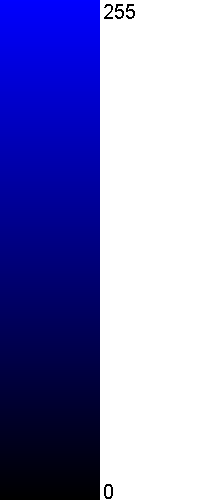

Supplement: Supplementary file 9 — Source Data for Expanded View [file EMBJ-41-e111608-s010.zip › SourceData/Figure_EV1/FEV1a/SARS2 0.1uM Tub/MetaData/SARS2 0.1uMch0LUT.png]

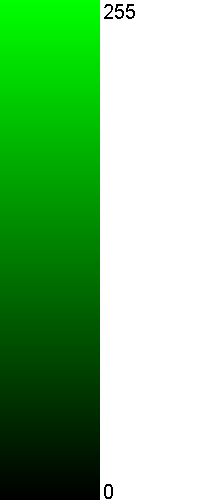

Supplement: Supplementary file 9 — Source Data for Expanded View [file EMBJ-41-e111608-s010.zip › SourceData/Figure_EV1/FEV1a/SARS2 0.1uM Tub/MetaData/SARS2 0.1uMch1LUT.png]

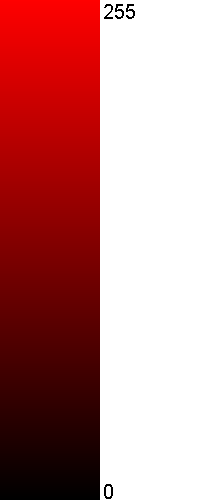

Supplement: Supplementary file 9 — Source Data for Expanded View [file EMBJ-41-e111608-s010.zip › SourceData/Figure_EV1/FEV1a/SARS2 0.1uM Tub/MetaData/SARS2 0.1uMch2LUT.png]

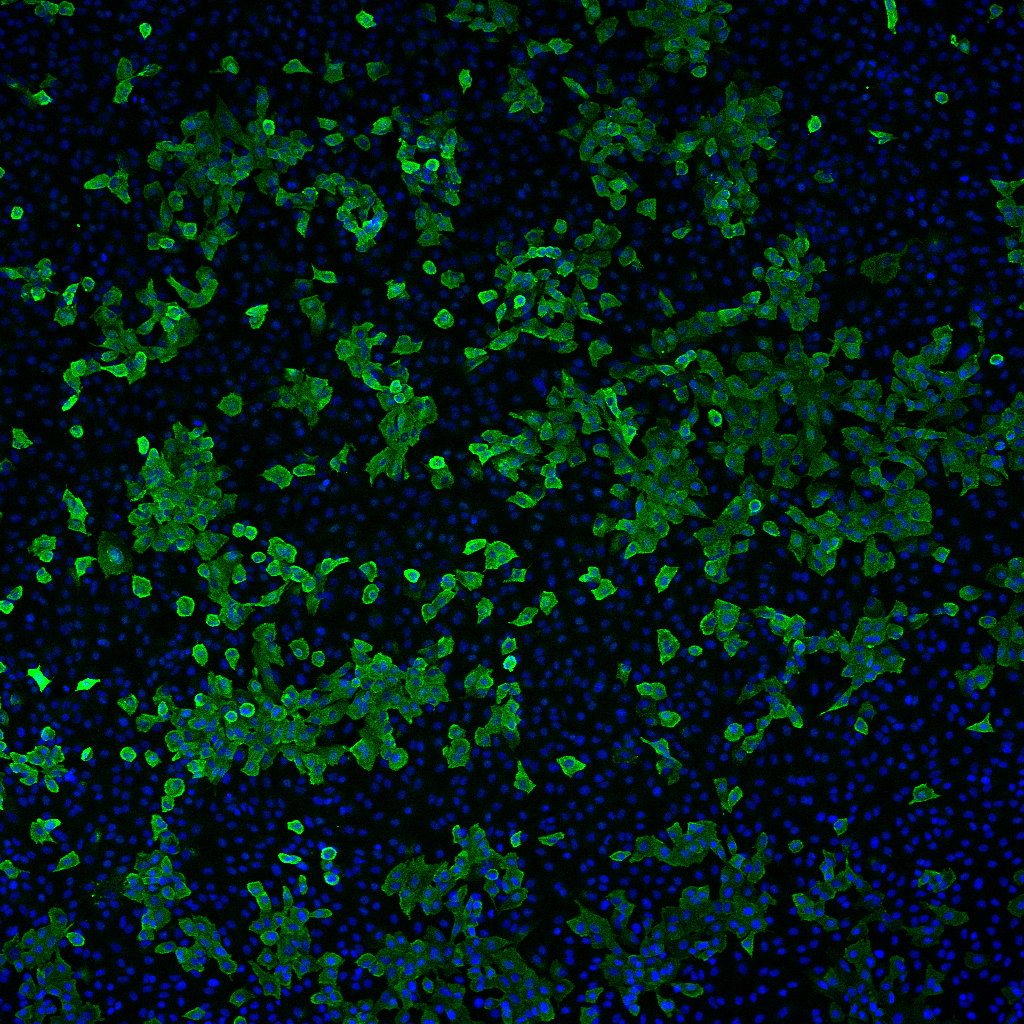

Supplement: Supplementary file 9 — Source Data for Expanded View [file EMBJ-41-e111608-s010.zip › SourceData/Figure_EV1/FEV1a/SARS2 0.1uM Tub/SARS2 0.1uM Tub (merge).tif]

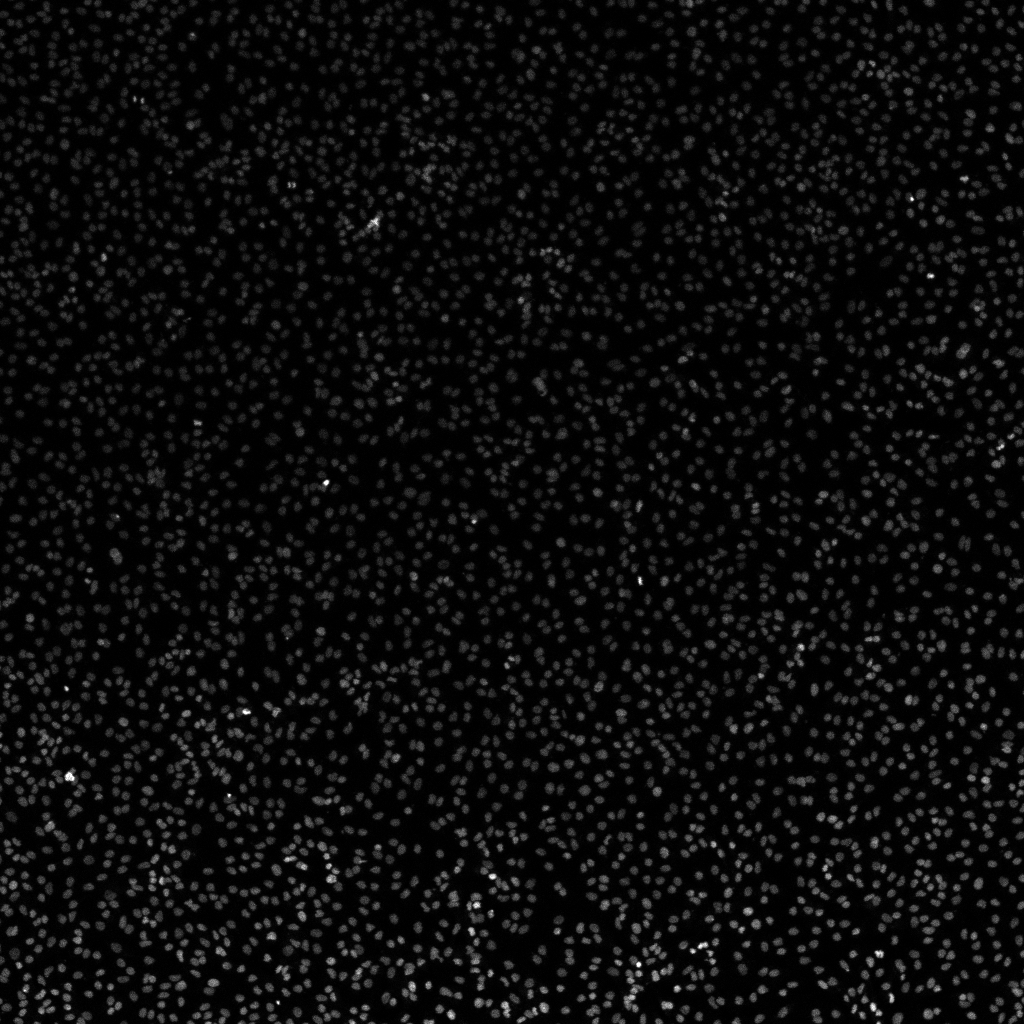

Supplement: Supplementary file 9 — Source Data for Expanded View [file EMBJ-41-e111608-s010.zip › SourceData/Figure_EV1/FEV1a/SARS2 0.1uM Tub/SARS2 0.1uM_DAPI.tif]

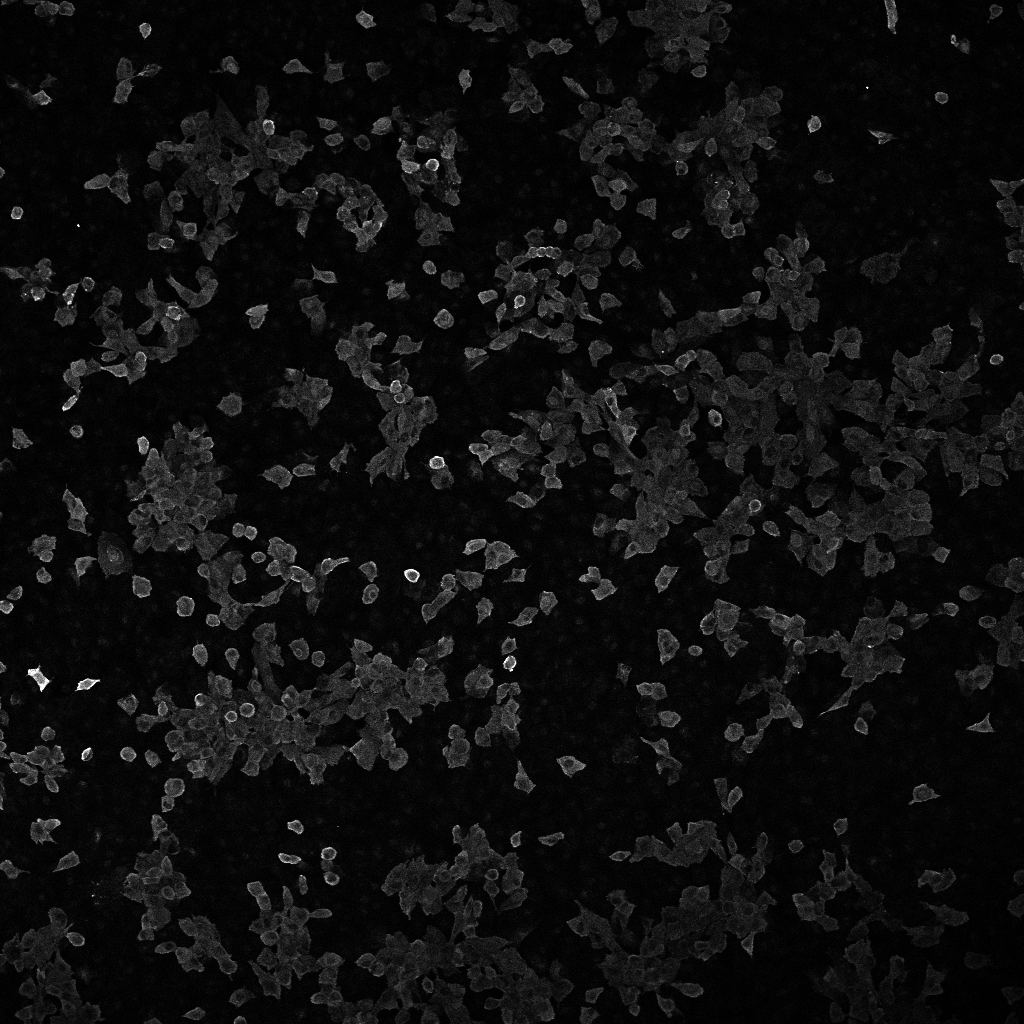

Supplement: Supplementary file 9 — Source Data for Expanded View [file EMBJ-41-e111608-s010.zip › SourceData/Figure_EV1/FEV1a/SARS2 0.1uM Tub/SARS2 0.1uM_N.tif]

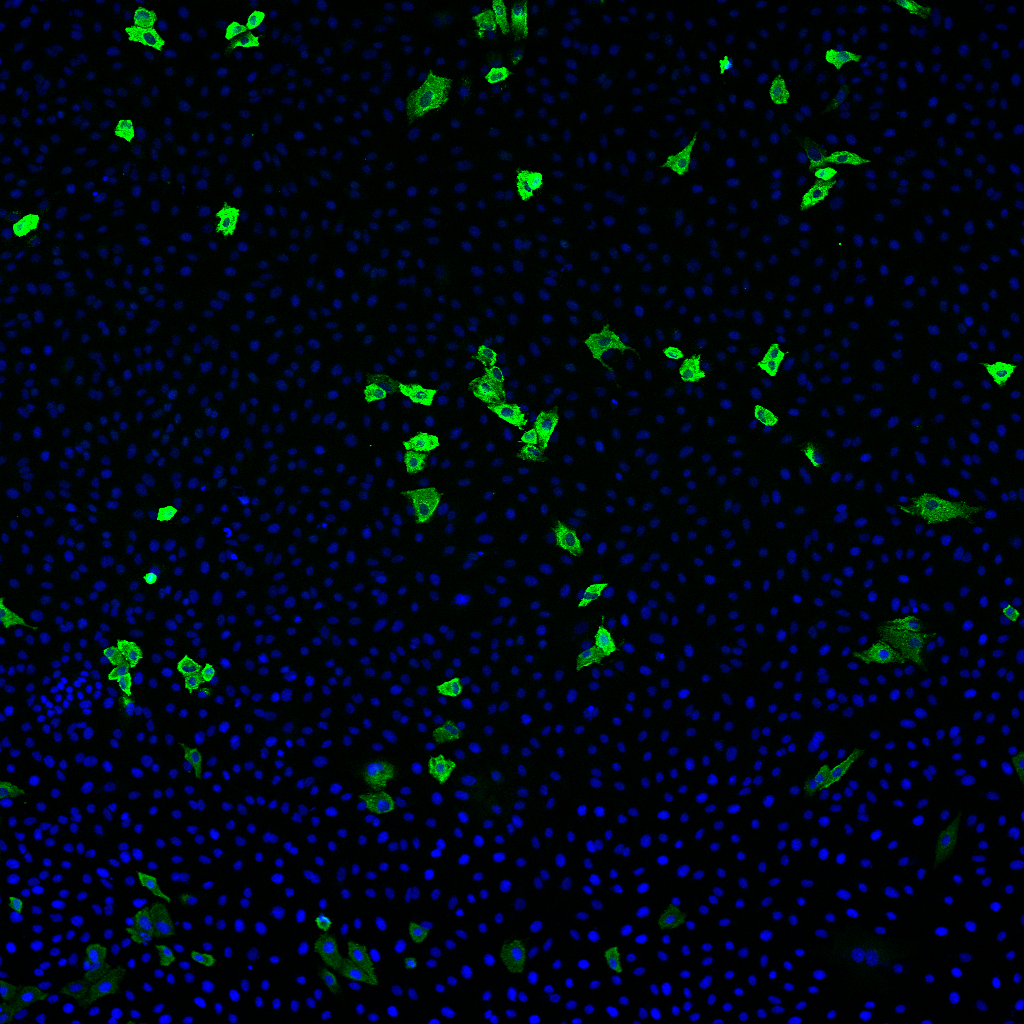

Supplement: Supplementary file 9 — Source Data for Expanded View [file EMBJ-41-e111608-s010.zip › SourceData/Figure_EV1/FEV1a/SARS2 1uM Tub/SARS2 1uM Tub (merge).tif]

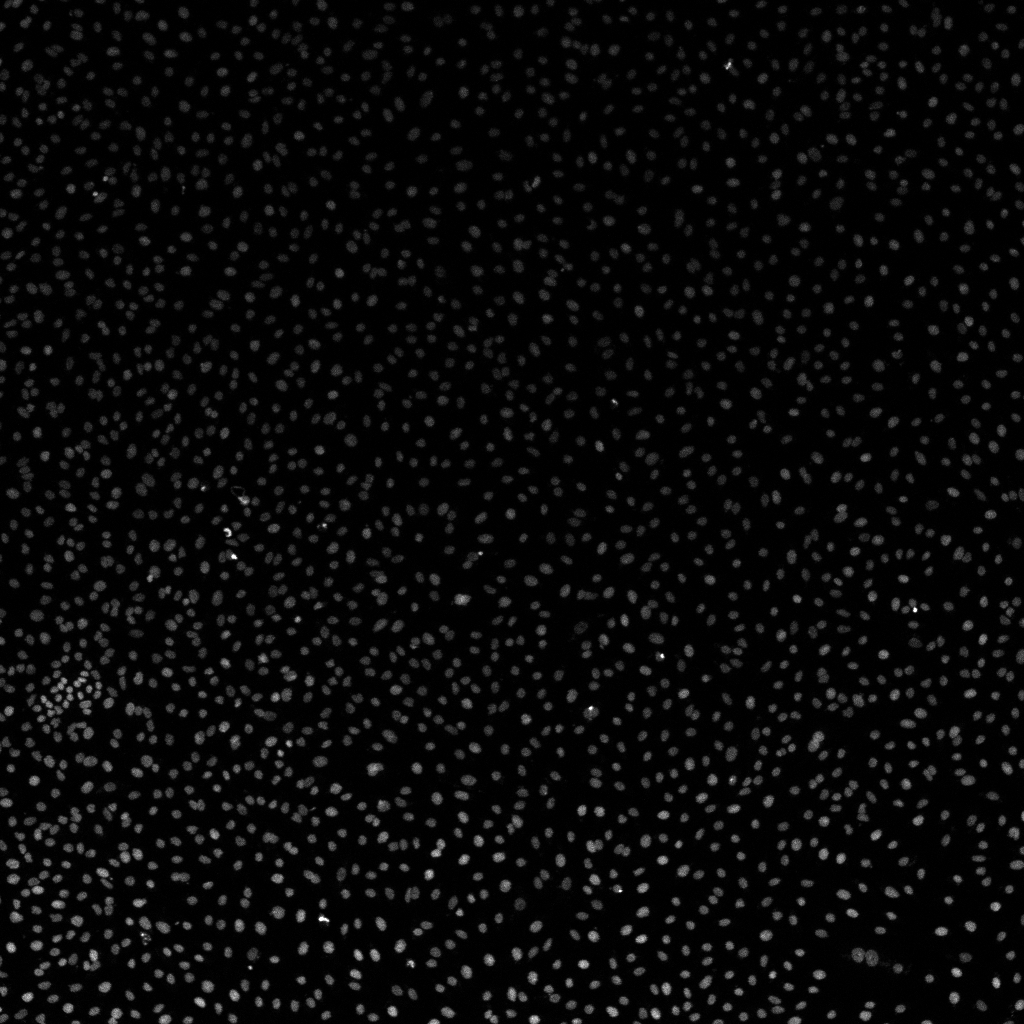

Supplement: Supplementary file 9 — Source Data for Expanded View [file EMBJ-41-e111608-s010.zip › SourceData/Figure_EV1/FEV1a/SARS2 1uM Tub/SARS2 1uM_DAPI.tif]

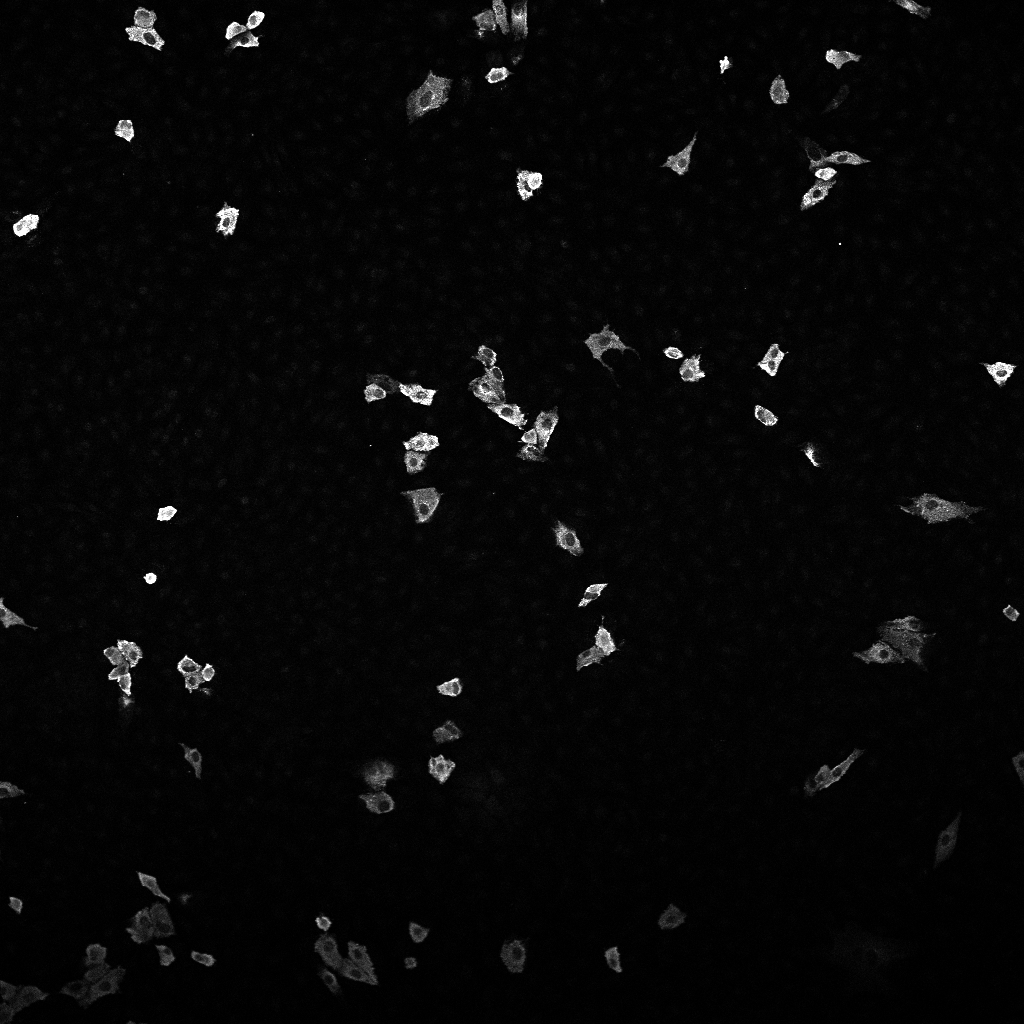

Supplement: Supplementary file 9 — Source Data for Expanded View [file EMBJ-41-e111608-s010.zip › SourceData/Figure_EV1/FEV1a/SARS2 1uM Tub/SARS2 1uM_N.tif]

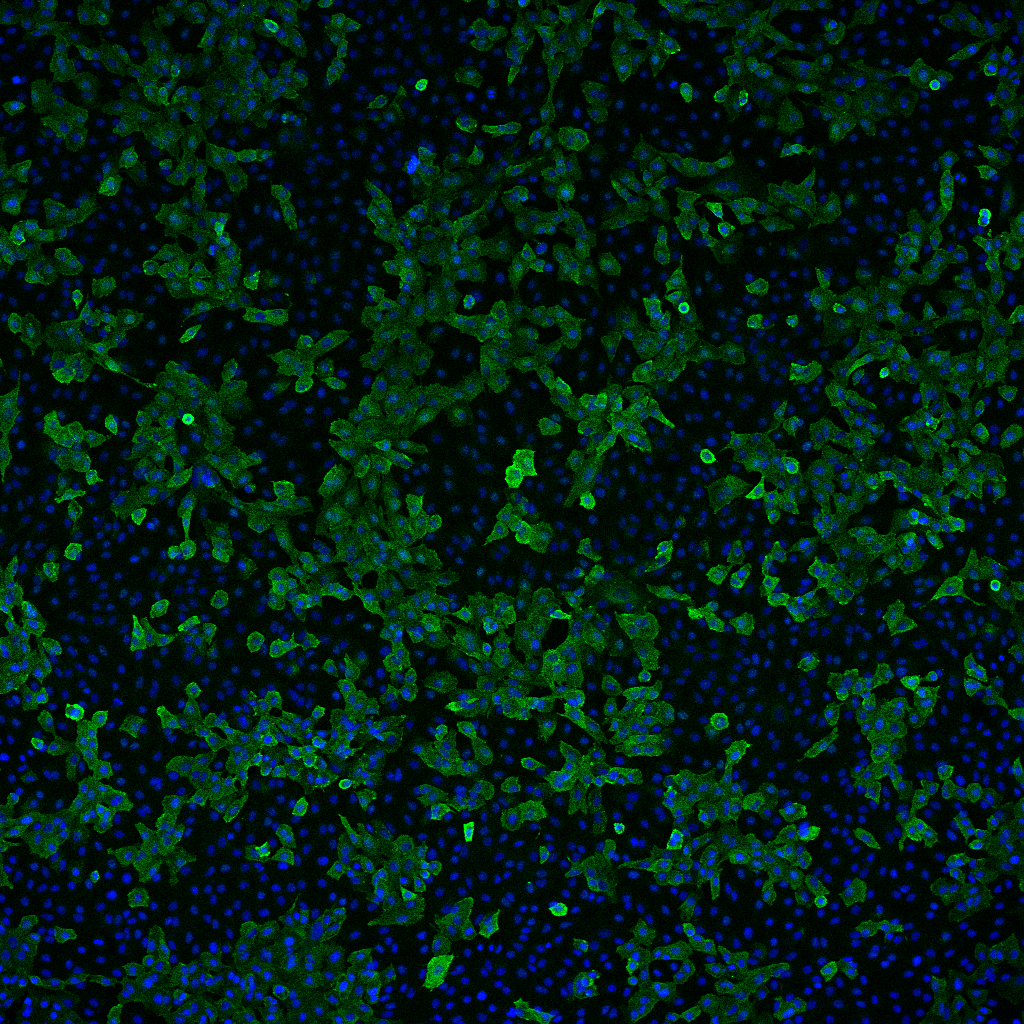

Supplement: Supplementary file 9 — Source Data for Expanded View [file EMBJ-41-e111608-s010.zip › SourceData/Figure_EV1/FEV1a/SARS2 DMSO/SARS2 DMSO (merge).tif]

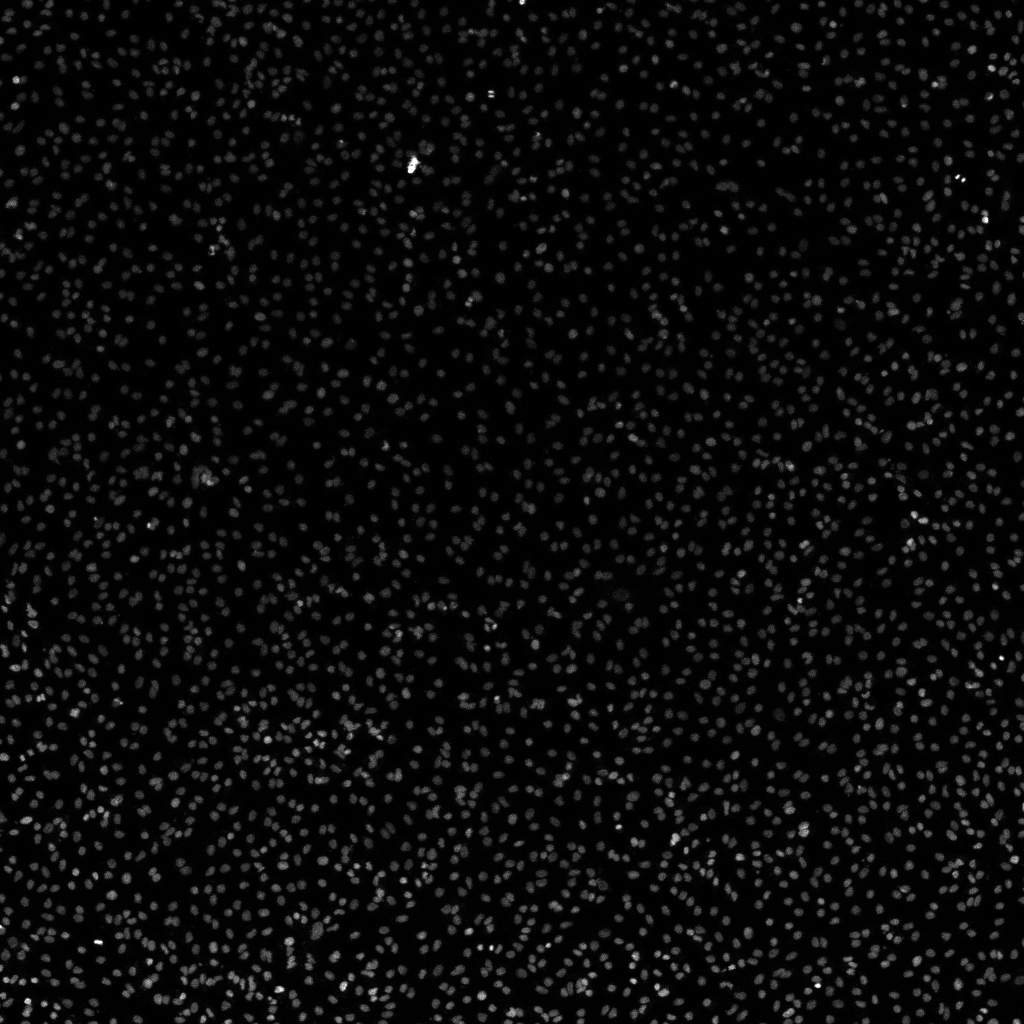

Supplement: Supplementary file 9 — Source Data for Expanded View [file EMBJ-41-e111608-s010.zip › SourceData/Figure_EV1/FEV1a/SARS2 DMSO/SARS2 DMSO_DAPI.tif]

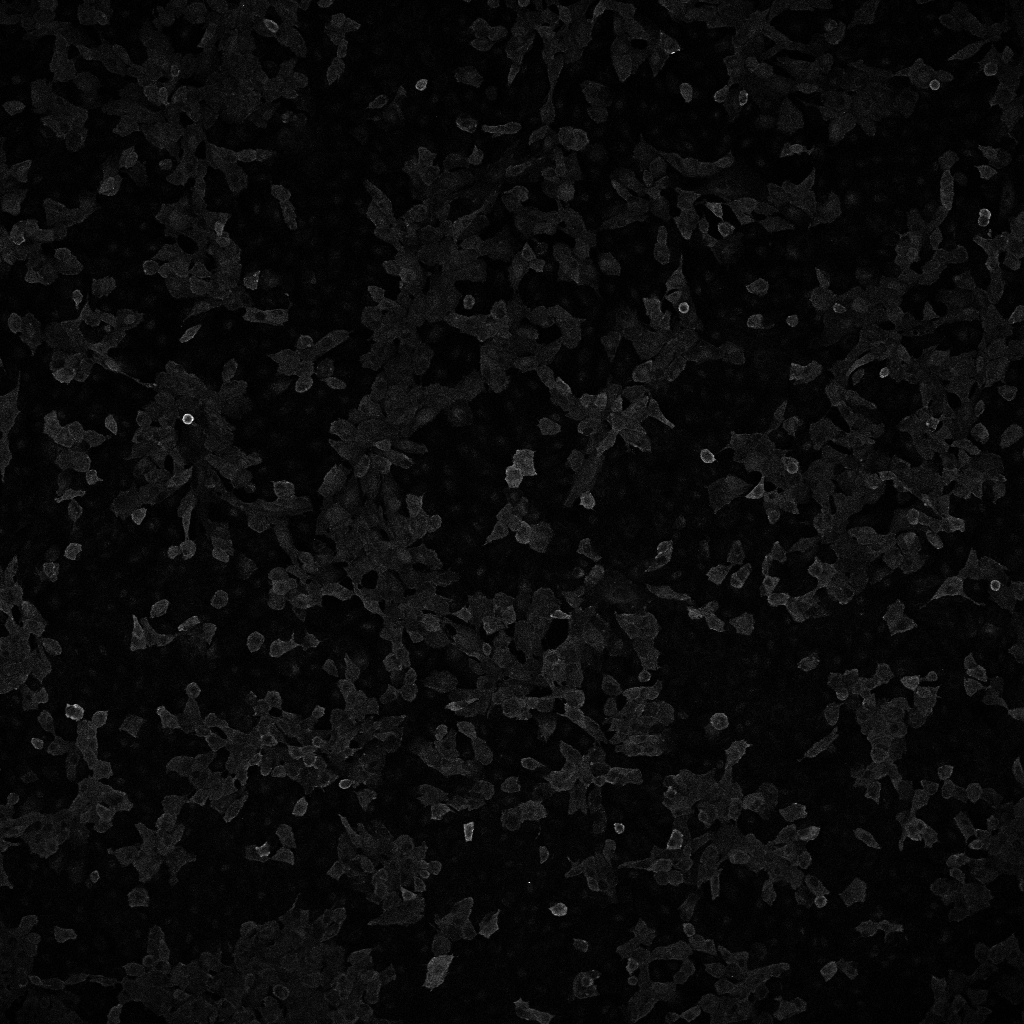

Supplement: Supplementary file 9 — Source Data for Expanded View [file EMBJ-41-e111608-s010.zip › SourceData/Figure_EV1/FEV1a/SARS2 DMSO/SARS2 DMSO_N.tif]

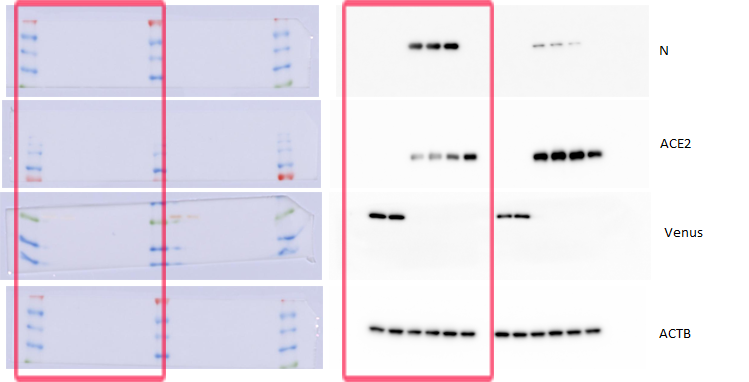

Supplement: Supplementary file 9 — Source Data for Expanded View [file EMBJ-41-e111608-s010.zip › SourceData/Figure_EV1/FEV1b/Figure_EV1b.png]

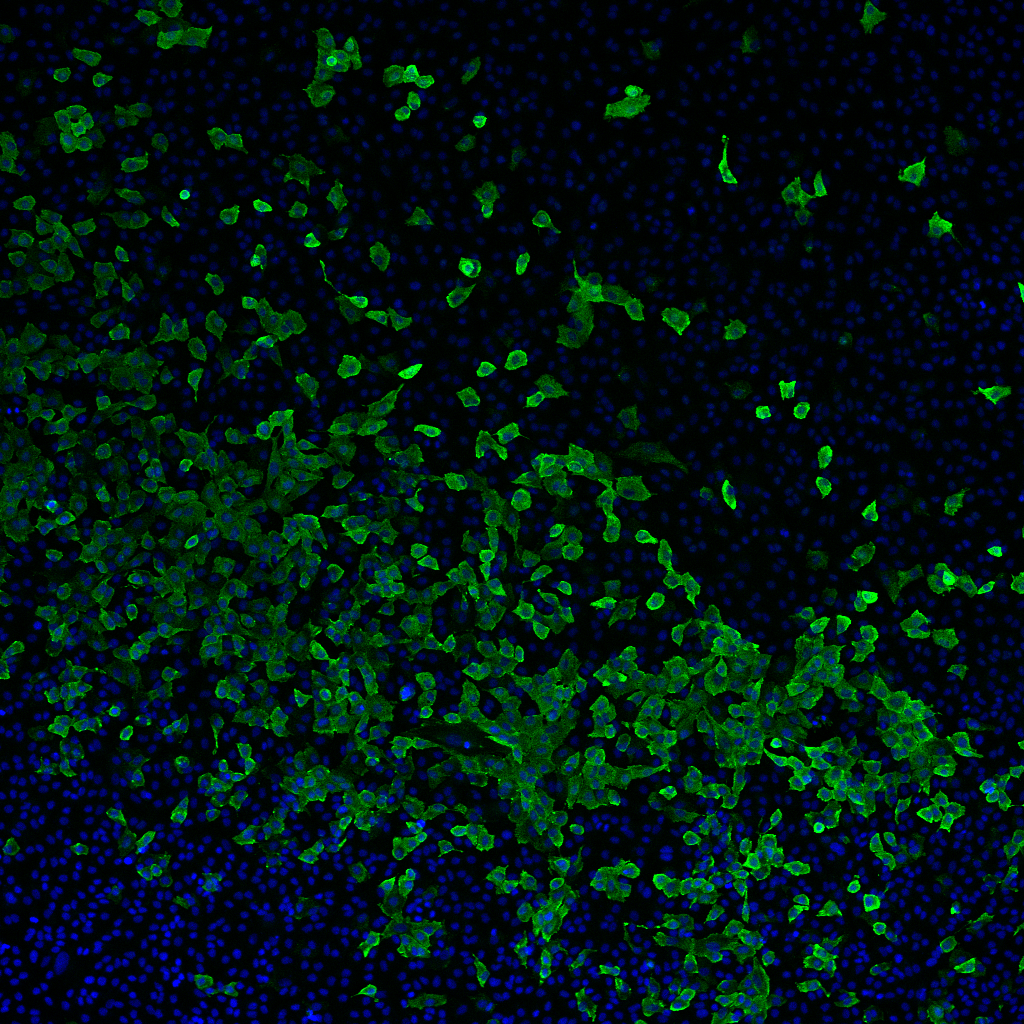

Supplement: Supplementary file 9 — Source Data for Expanded View [file EMBJ-41-e111608-s010.zip › SourceData/Figure_EV1/FEV1c/SARS1 0.1uM Tub/SARS1 0.1uM Tub (merge).tif]

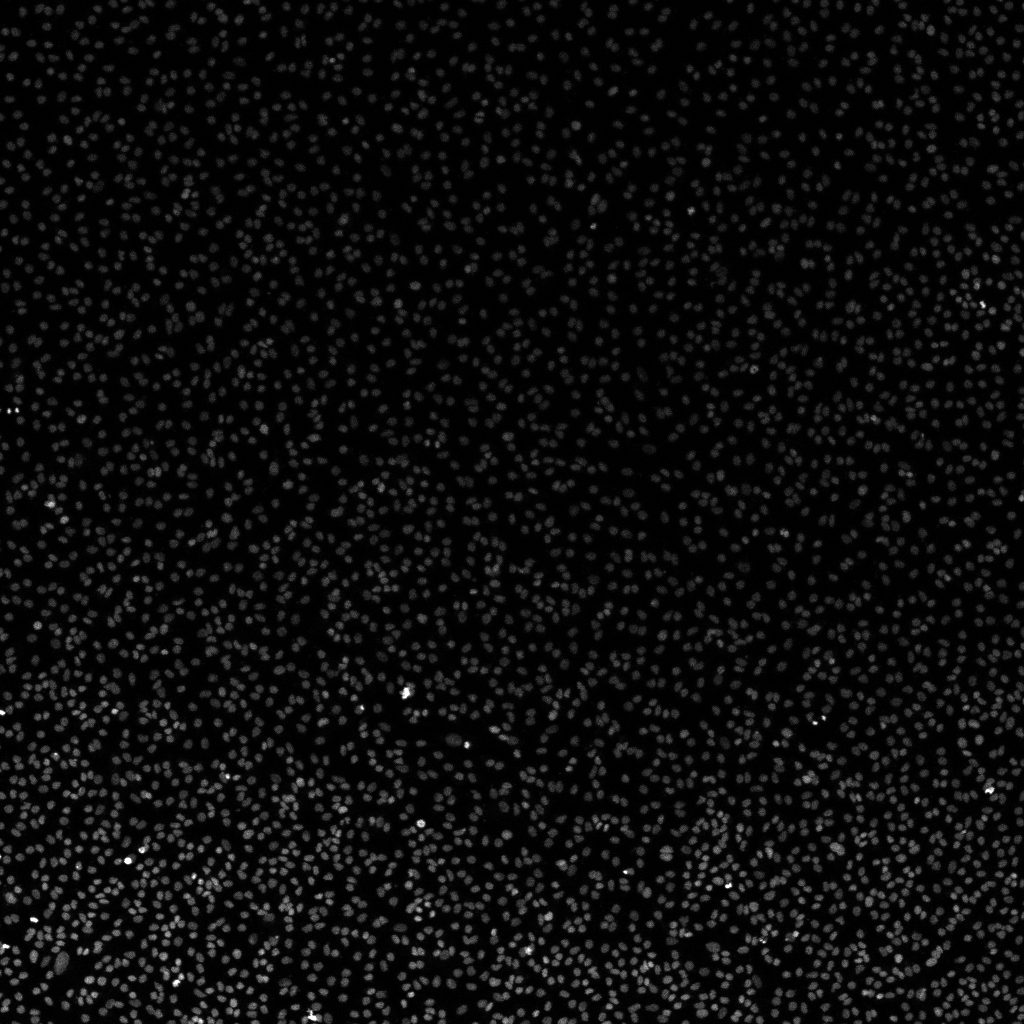

Supplement: Supplementary file 9 — Source Data for Expanded View [file EMBJ-41-e111608-s010.zip › SourceData/Figure_EV1/FEV1c/SARS1 0.1uM Tub/SARS1 0.1uM_DAPI.tif]

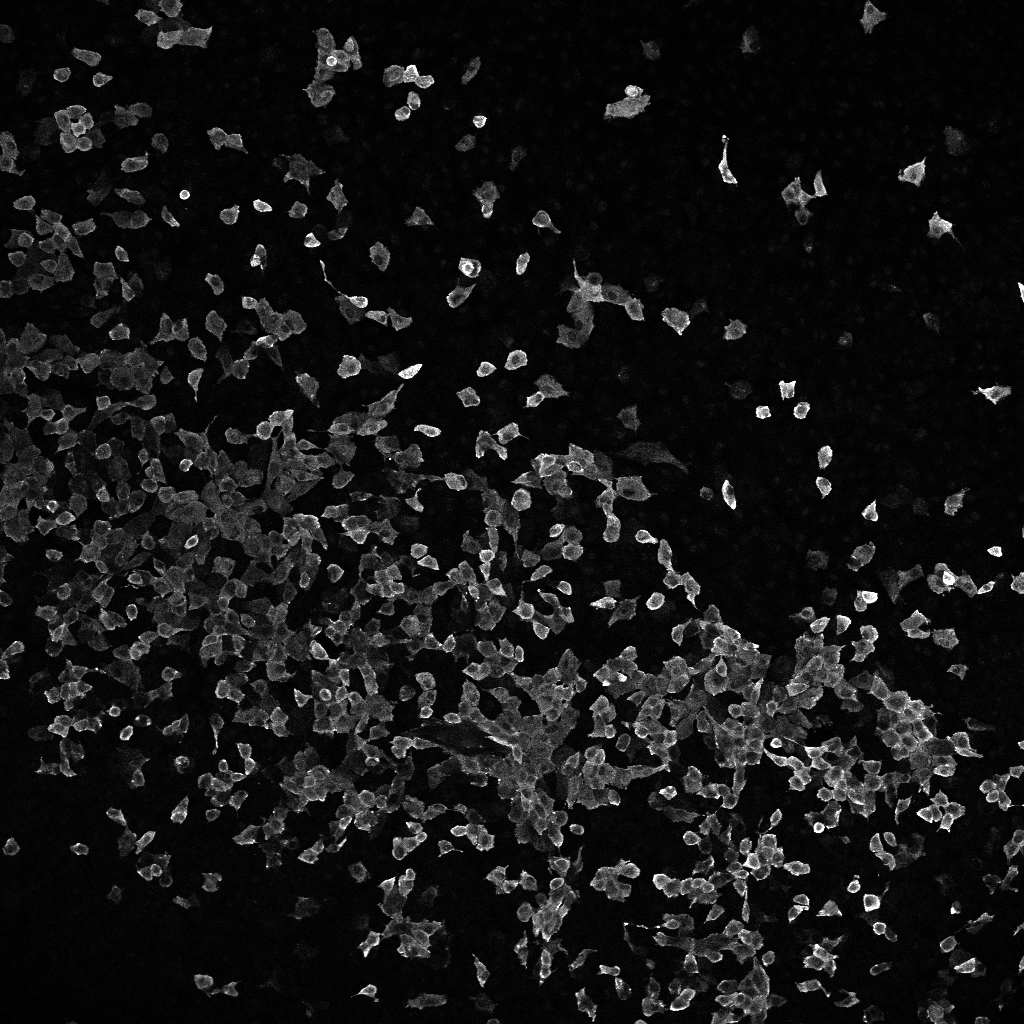

Supplement: Supplementary file 9 — Source Data for Expanded View [file EMBJ-41-e111608-s010.zip › SourceData/Figure_EV1/FEV1c/SARS1 0.1uM Tub/SARS1 0.1uM_N.tif]

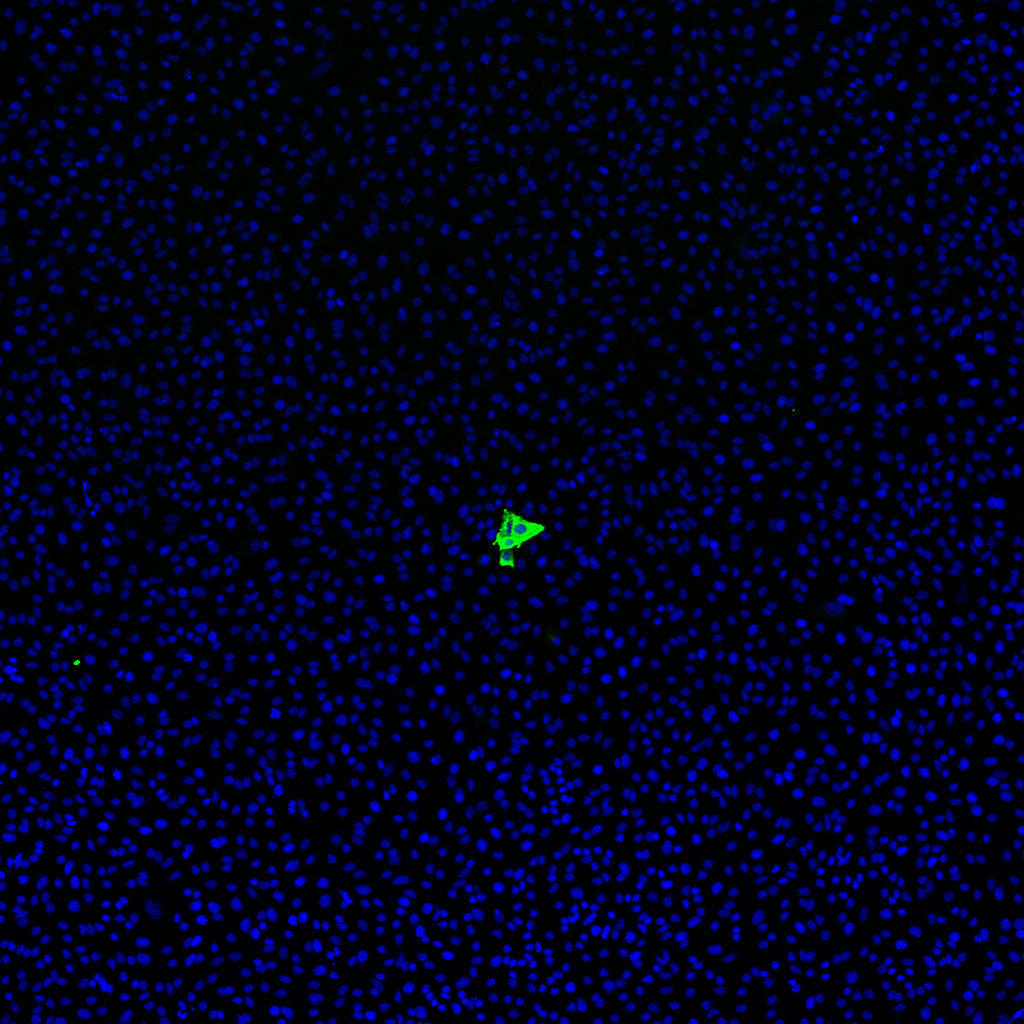

Supplement: Supplementary file 9 — Source Data for Expanded View [file EMBJ-41-e111608-s010.zip › SourceData/Figure_EV1/FEV1c/SARS1 1uM Tub/SARS1 1uM Tub (merge).tif]

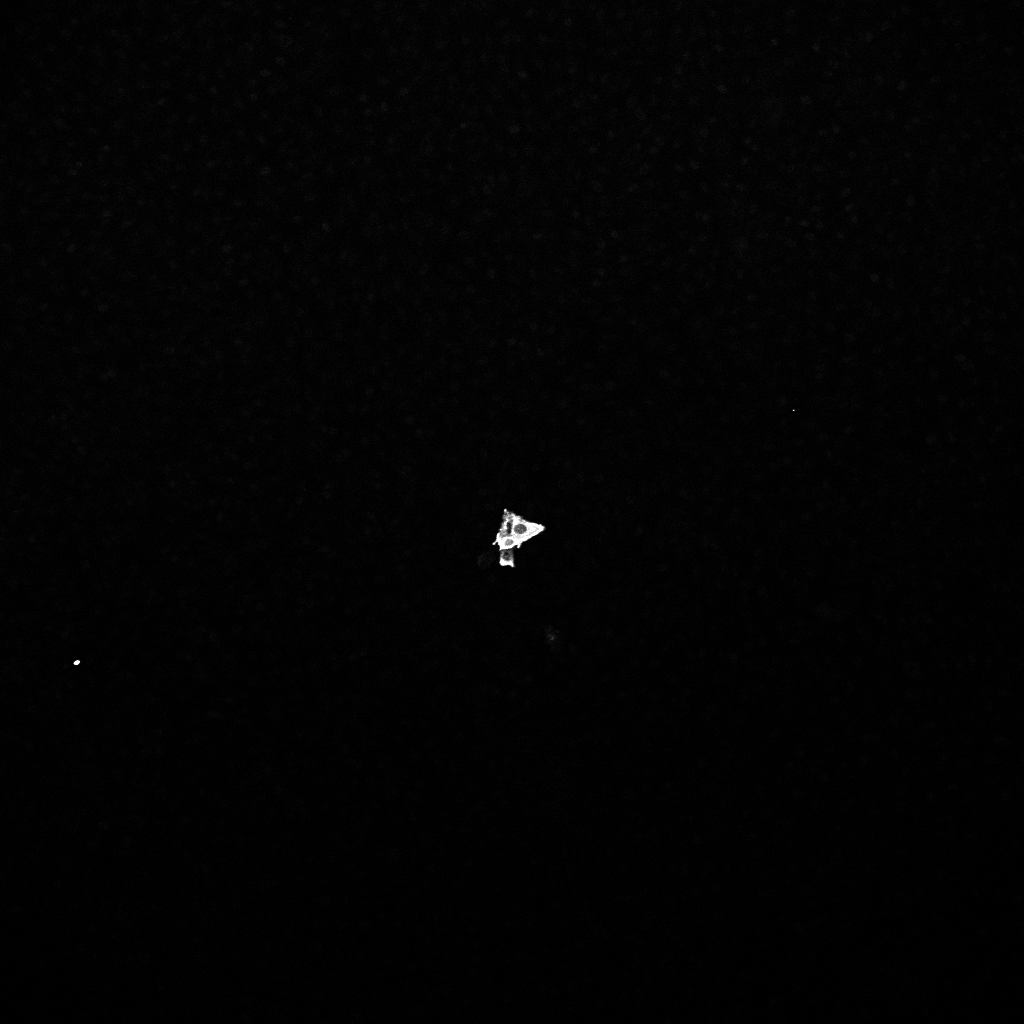

Supplement: Supplementary file 9 — Source Data for Expanded View [file EMBJ-41-e111608-s010.zip › SourceData/Figure_EV1/FEV1c/SARS1 1uM Tub/SARS1 1uM_ch01.tif]

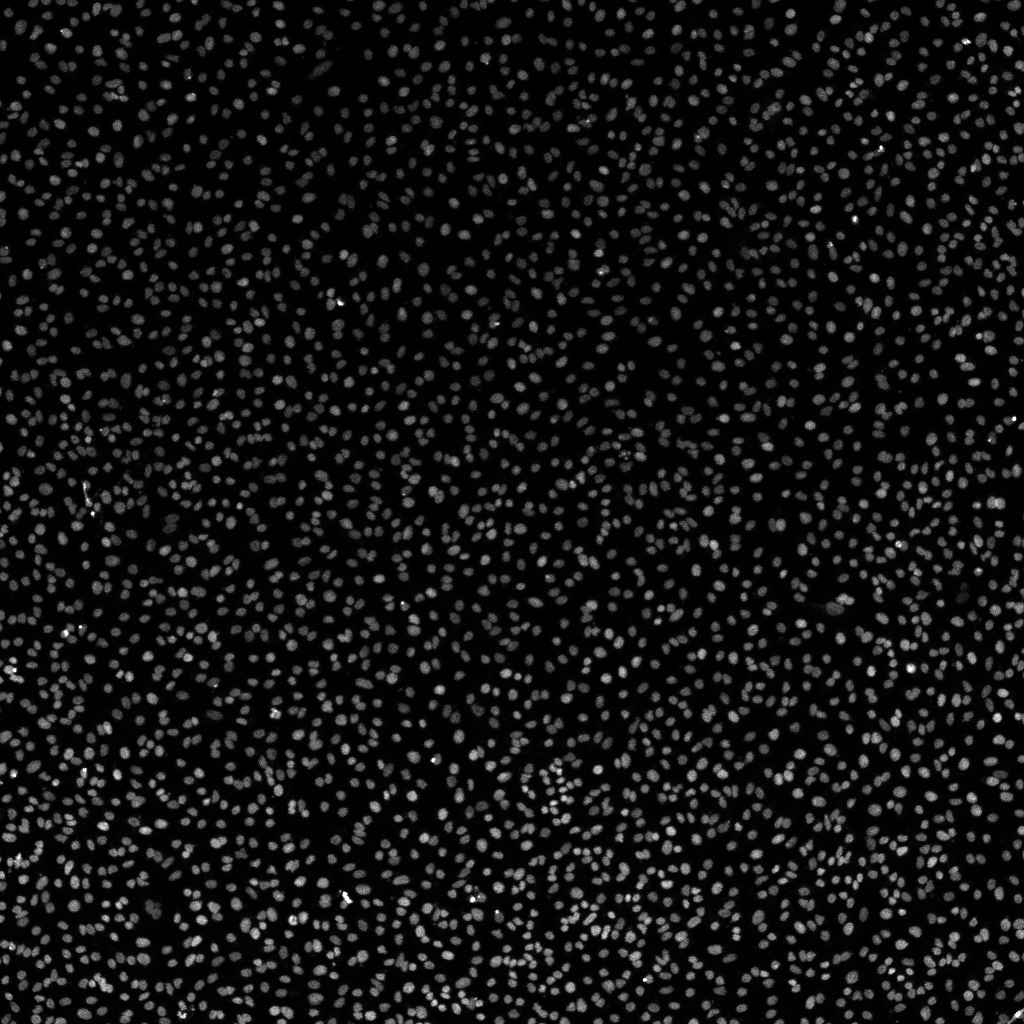

Supplement: Supplementary file 9 — Source Data for Expanded View [file EMBJ-41-e111608-s010.zip › SourceData/Figure_EV1/FEV1c/SARS1 1uM Tub/SARS1 1uM_N.tif]

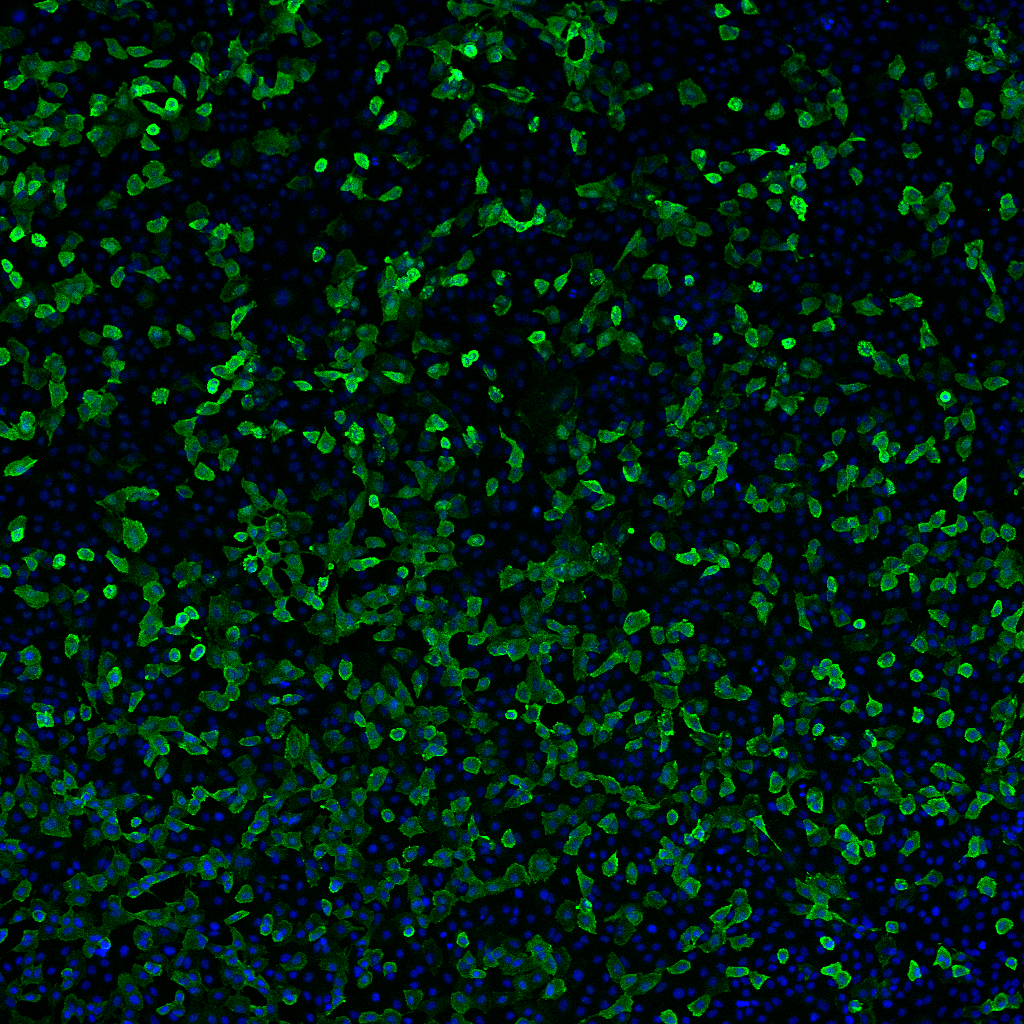

Supplement: Supplementary file 9 — Source Data for Expanded View [file EMBJ-41-e111608-s010.zip › SourceData/Figure_EV1/FEV1c/SARS1 DMSO/SARS1 DMSO (merge).tif]

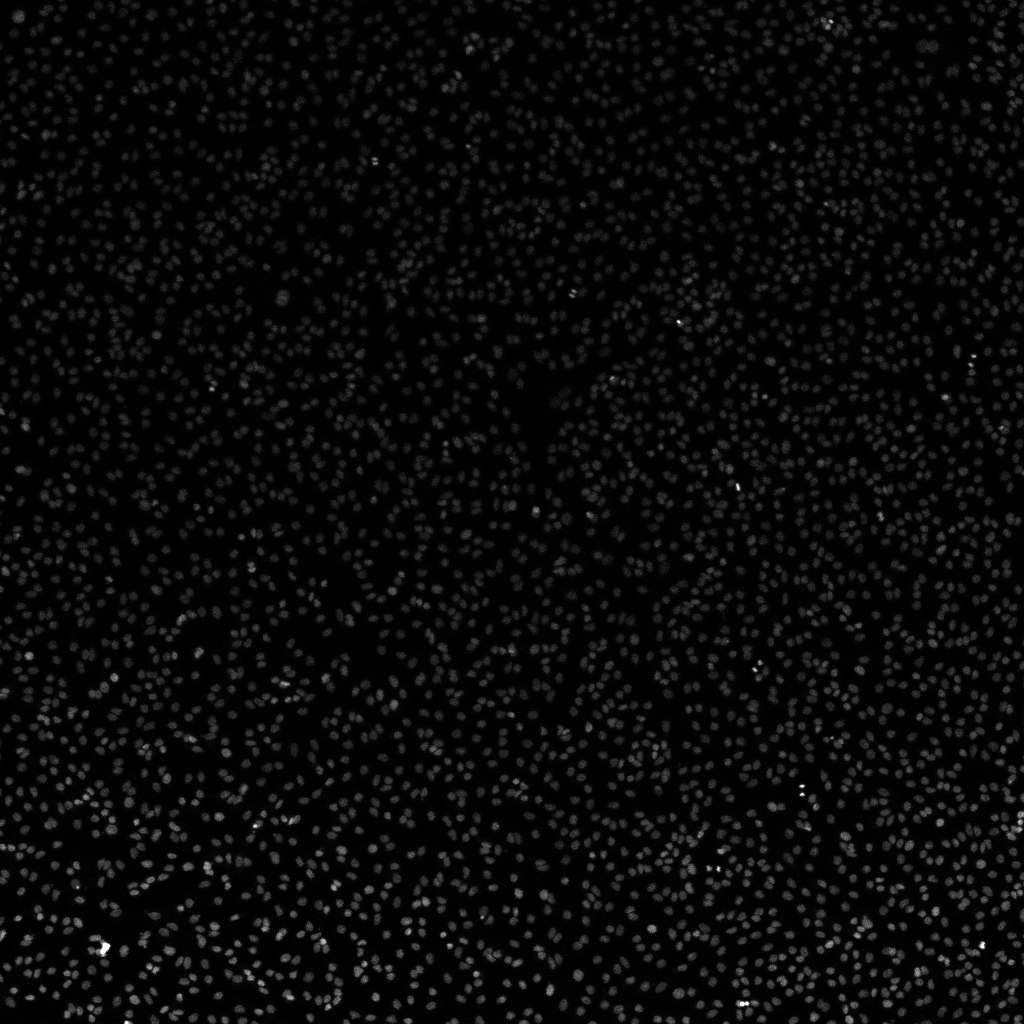

Supplement: Supplementary file 9 — Source Data for Expanded View [file EMBJ-41-e111608-s010.zip › SourceData/Figure_EV1/FEV1c/SARS1 DMSO/SARS1 DMSO_DAPI.tif]

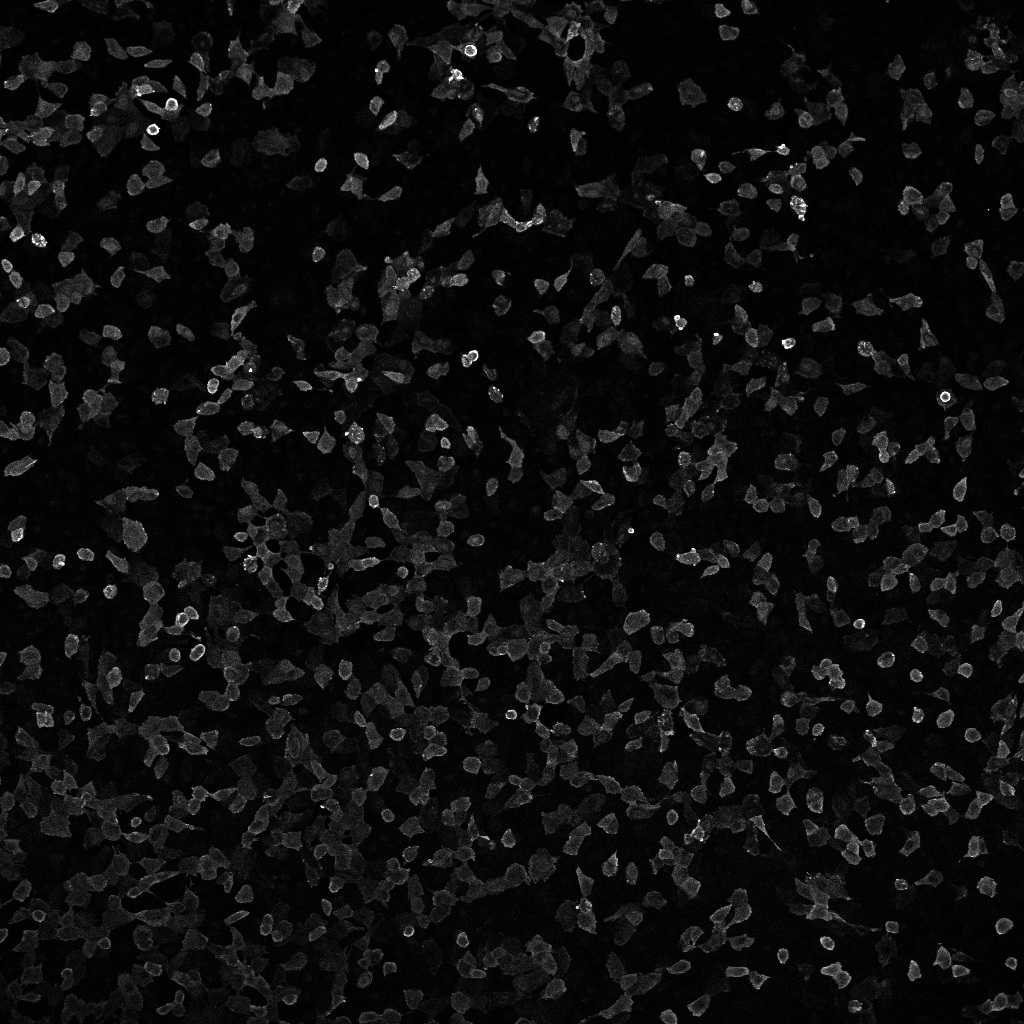

Supplement: Supplementary file 9 — Source Data for Expanded View [file EMBJ-41-e111608-s010.zip › SourceData/Figure_EV1/FEV1c/SARS1 DMSO/SARS1 DMSO_N.tif]

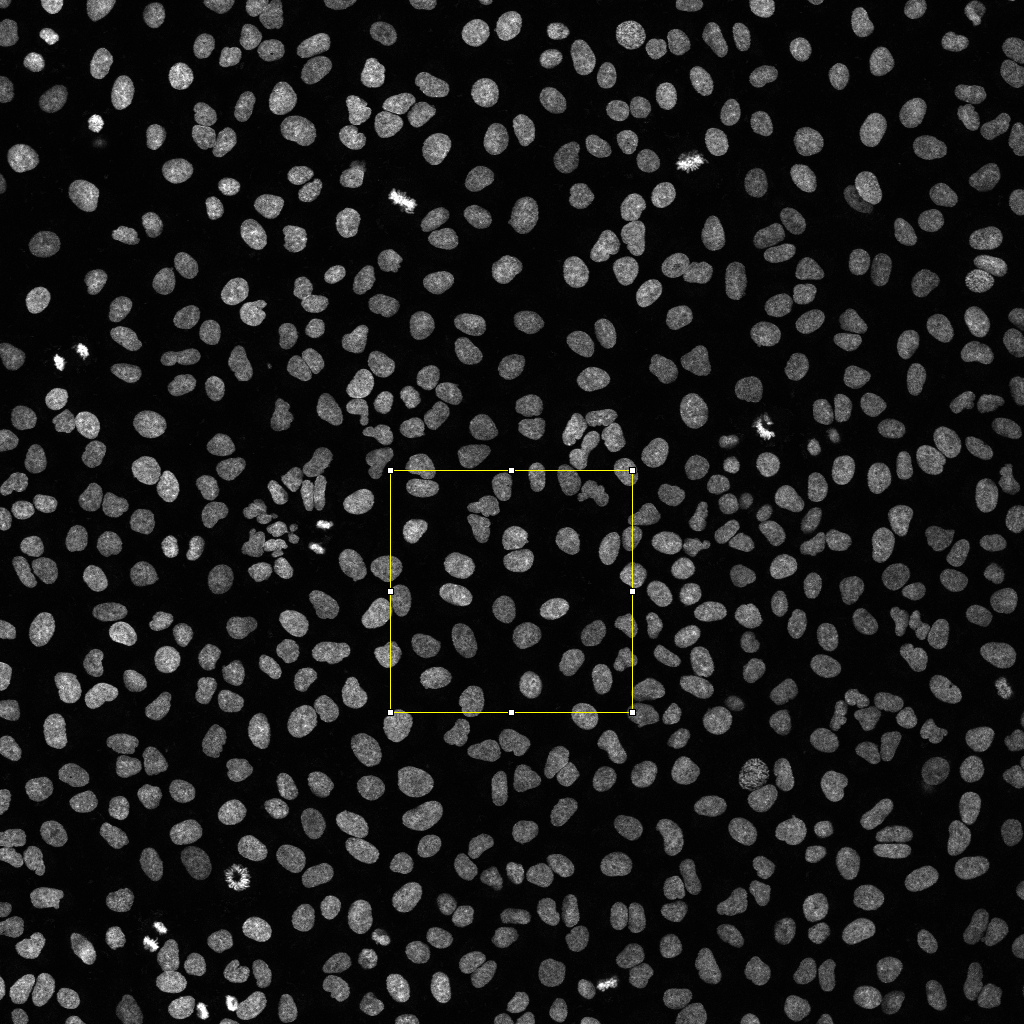

Supplement: Supplementary file 9 — Source Data for Expanded View [file EMBJ-41-e111608-s010.zip › SourceData/Figure_EV1/FEV1h/A549 DAPI ROI.tif]

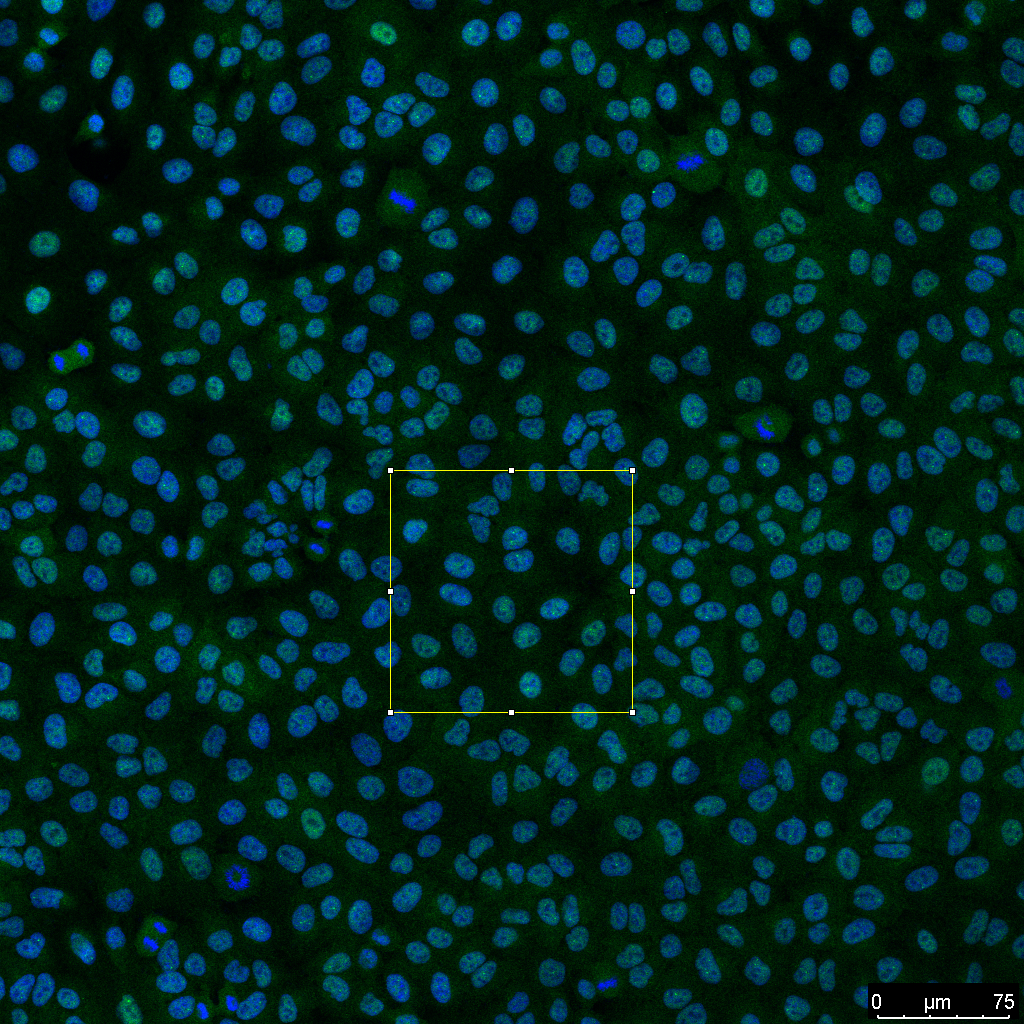

Supplement: Supplementary file 9 — Source Data for Expanded View [file EMBJ-41-e111608-s010.zip › SourceData/Figure_EV1/FEV1h/A549 merge ROI.tif]

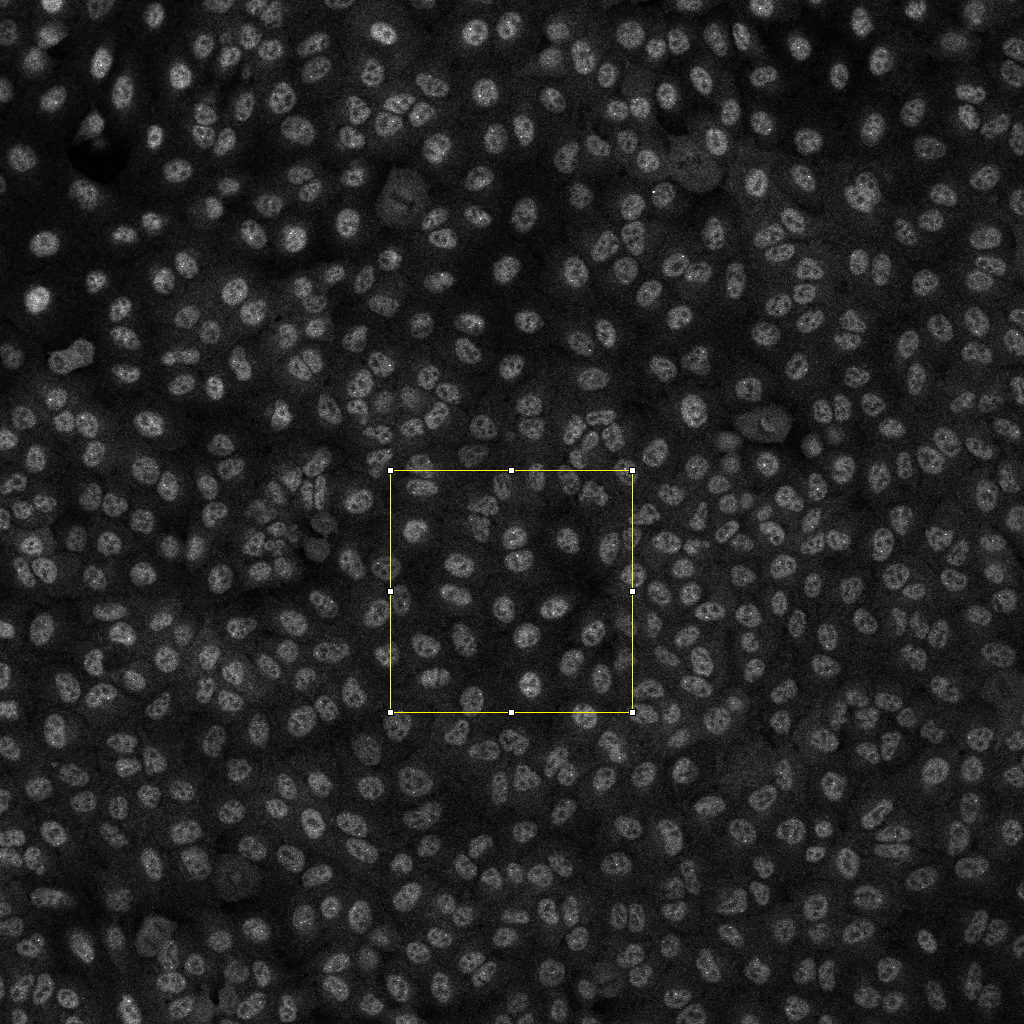

Supplement: Supplementary file 9 — Source Data for Expanded View [file EMBJ-41-e111608-s010.zip › SourceData/Figure_EV1/FEV1h/A549 MTr1 ROI.tif]

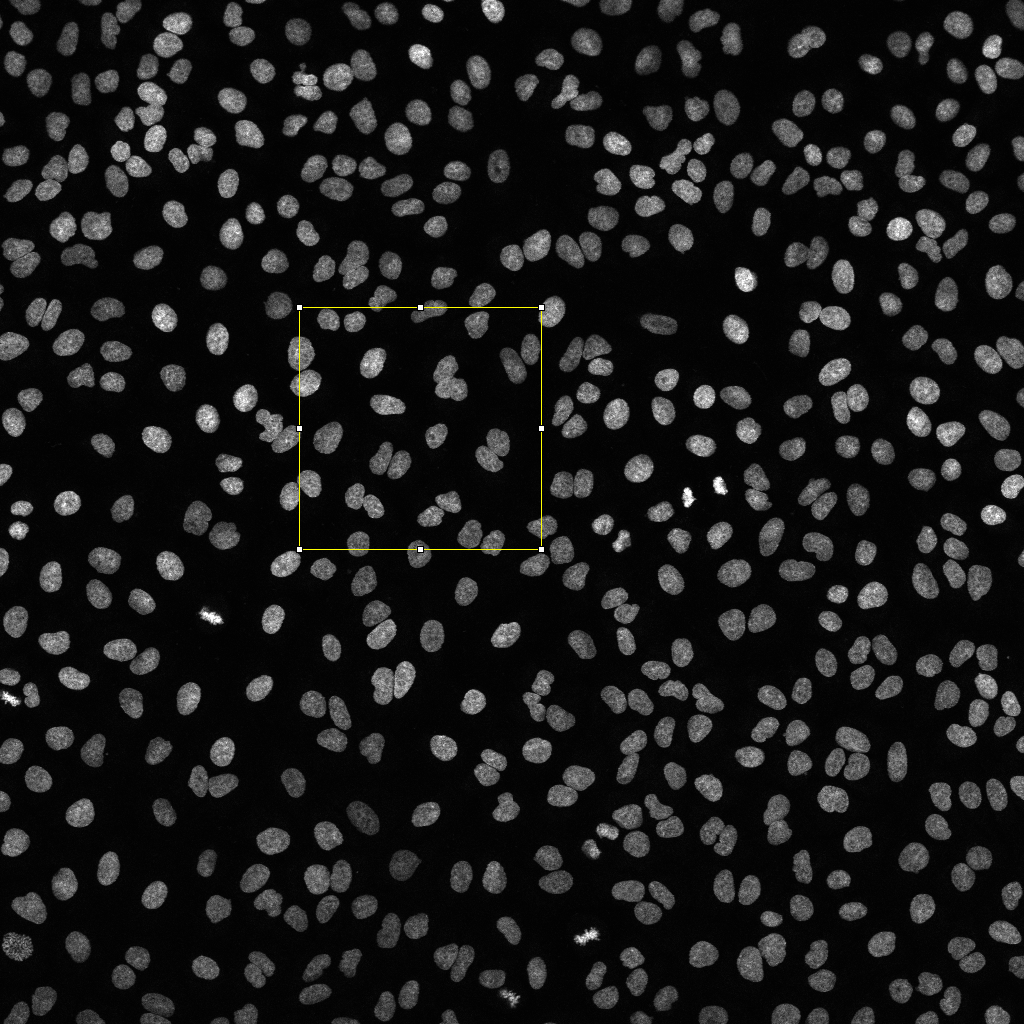

Supplement: Supplementary file 9 — Source Data for Expanded View [file EMBJ-41-e111608-s010.zip › SourceData/Figure_EV1/FEV1h/A549+IFN DAPI ROI.tif]

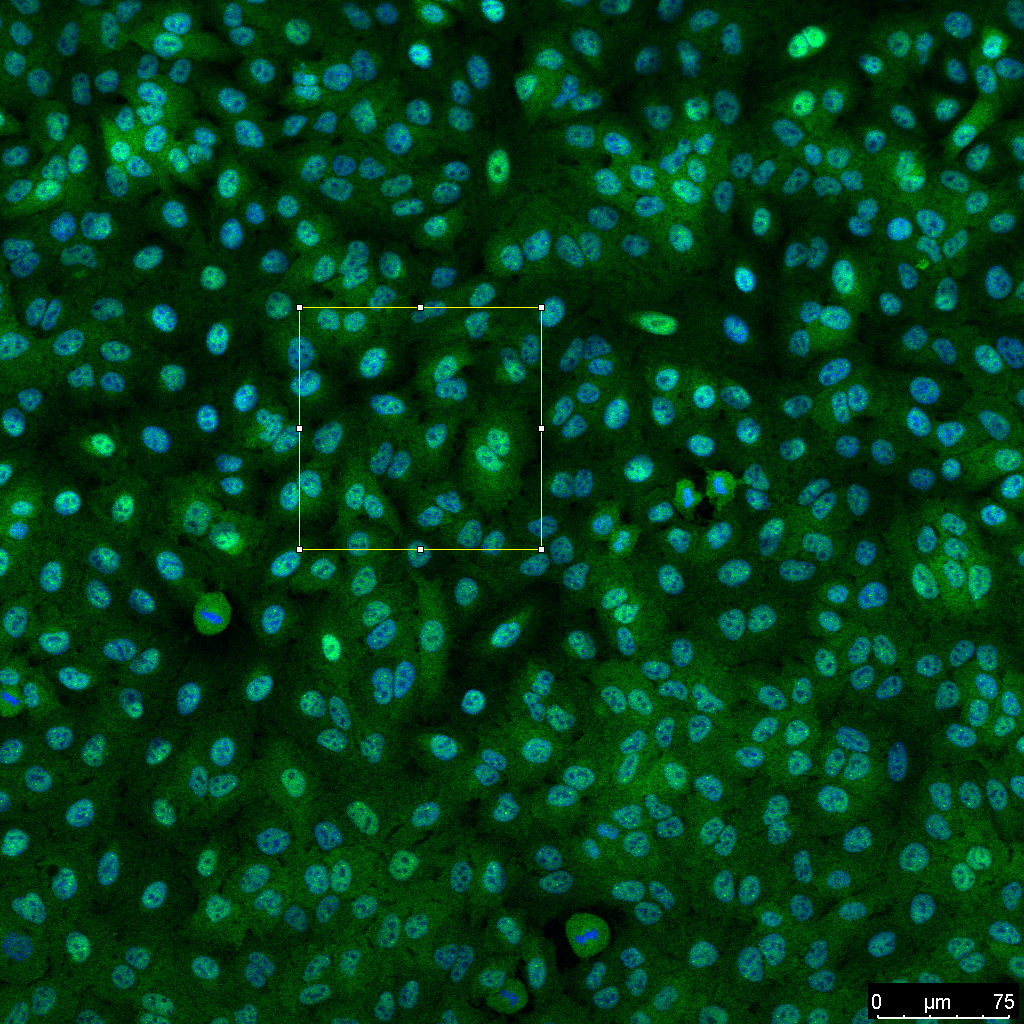

Supplement: Supplementary file 9 — Source Data for Expanded View [file EMBJ-41-e111608-s010.zip › SourceData/Figure_EV1/FEV1h/A549+IFN merge ROI.tif]

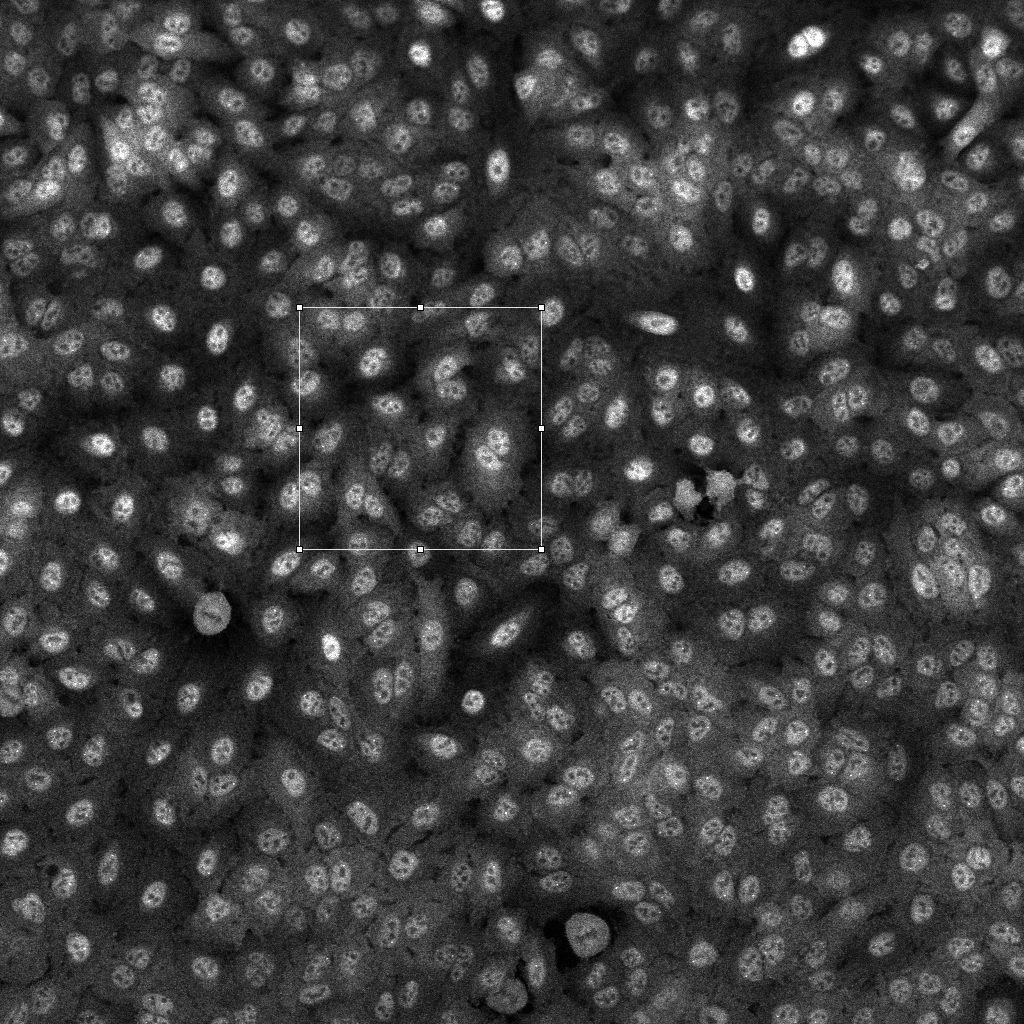

Supplement: Supplementary file 9 — Source Data for Expanded View [file EMBJ-41-e111608-s010.zip › SourceData/Figure_EV1/FEV1h/A549+IFN MTr1 ROI.tif]

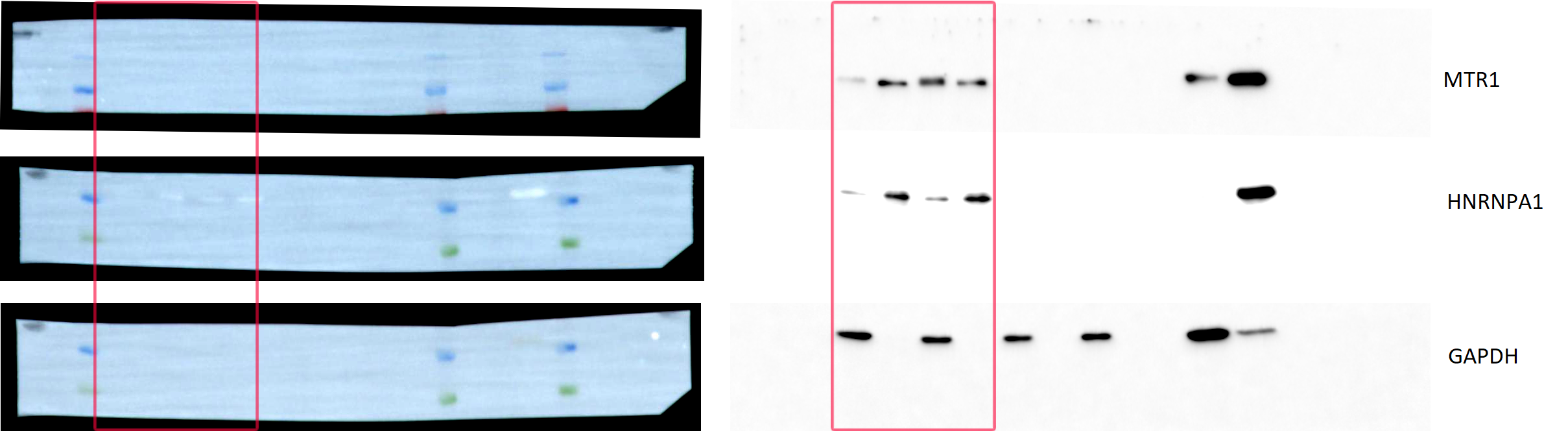

Supplement: Supplementary file 9 — Source Data for Expanded View [file EMBJ-41-e111608-s010.zip › SourceData/Figure_EV1/FEV1i/Figure_EV1i.png]

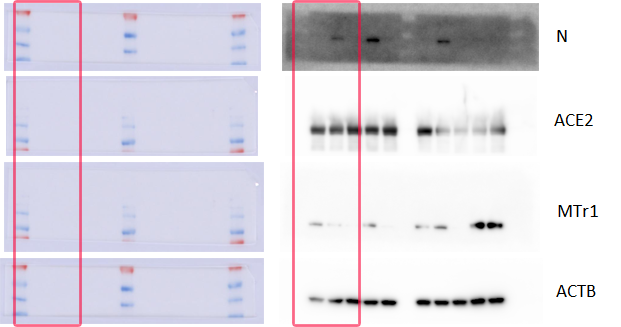

Supplement: Supplementary file 9 — Source Data for Expanded View [file EMBJ-41-e111608-s010.zip › SourceData/Figure_EV1/FEV1j/Figure_EV1j.png]

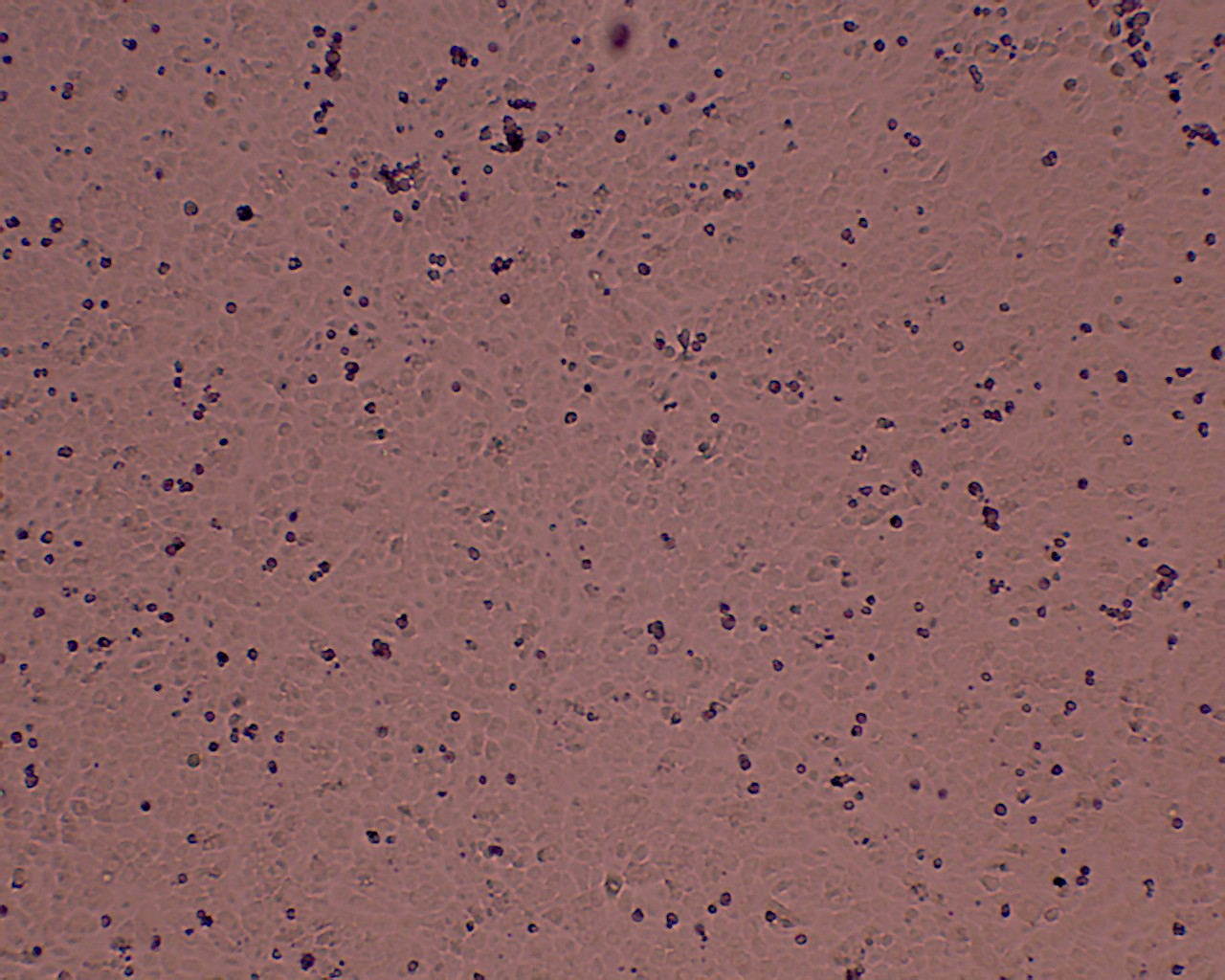

Supplement: Supplementary file 9 — Source Data for Expanded View [file EMBJ-41-e111608-s010.zip › SourceData/Figure_EV1/FEV1k/ko.tif]

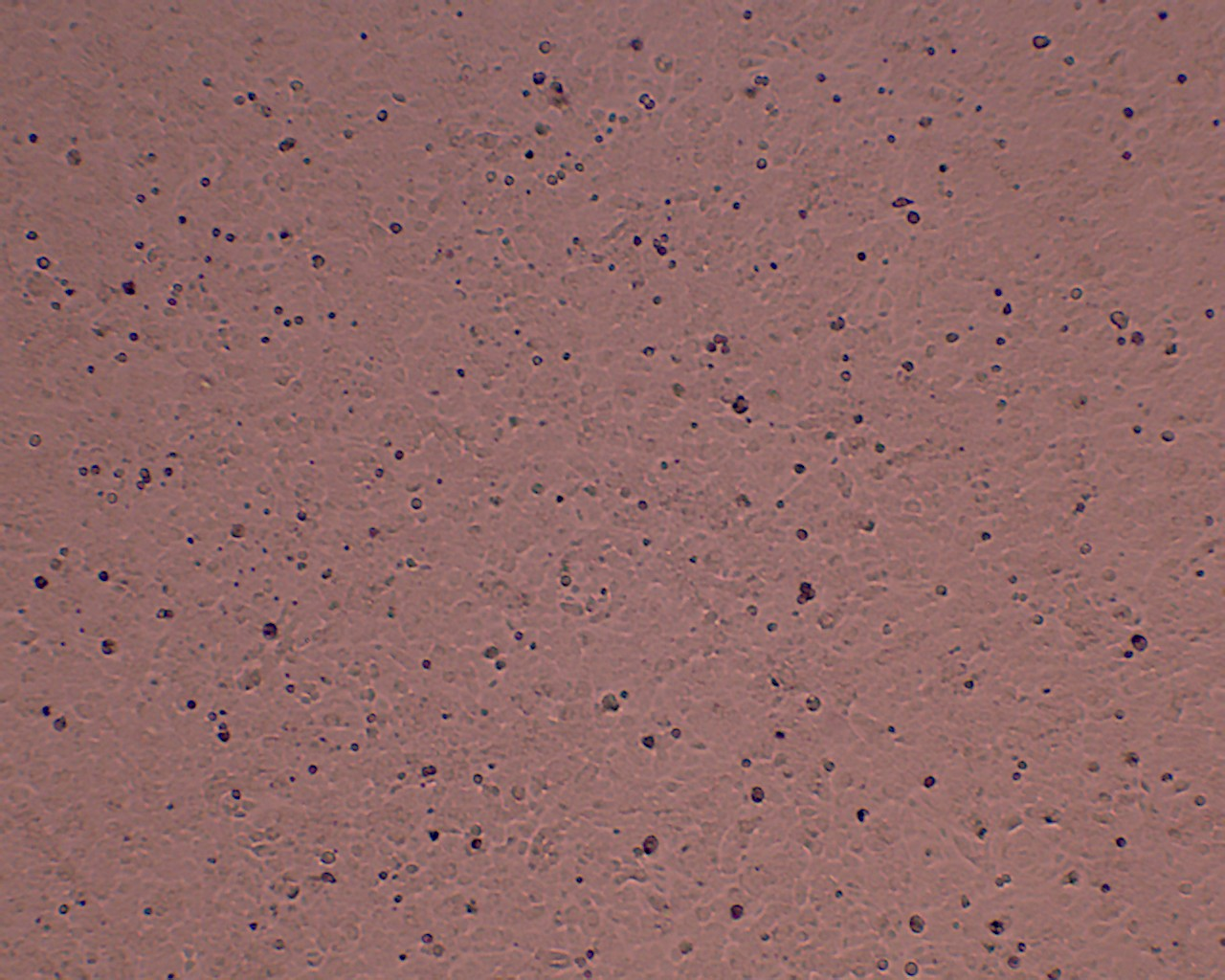

Supplement: Supplementary file 9 — Source Data for Expanded View [file EMBJ-41-e111608-s010.zip › SourceData/Figure_EV1/FEV1k/wt mock.tif]

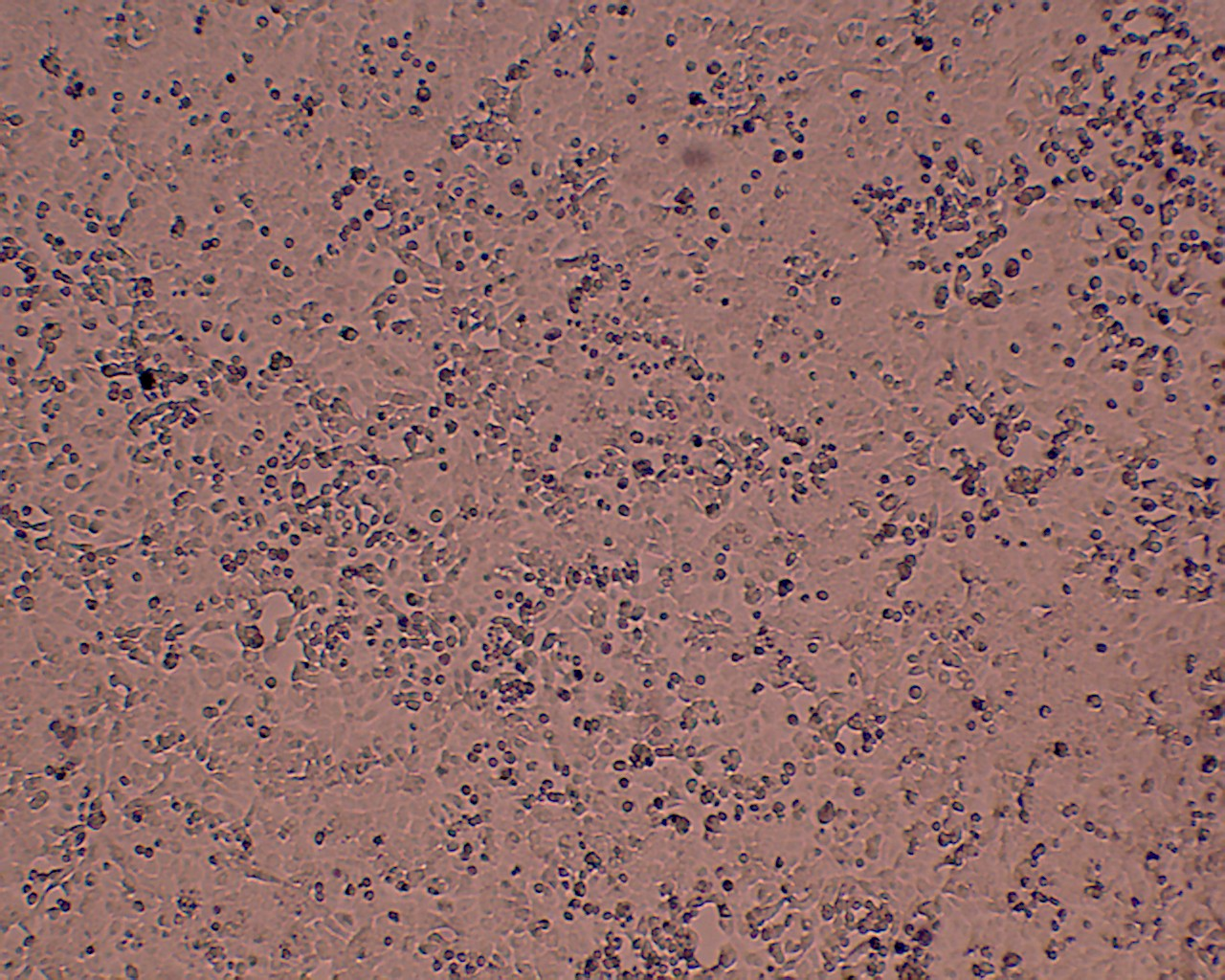

Supplement: Supplementary file 9 — Source Data for Expanded View [file EMBJ-41-e111608-s010.zip › SourceData/Figure_EV1/FEV1k/wt.tif]

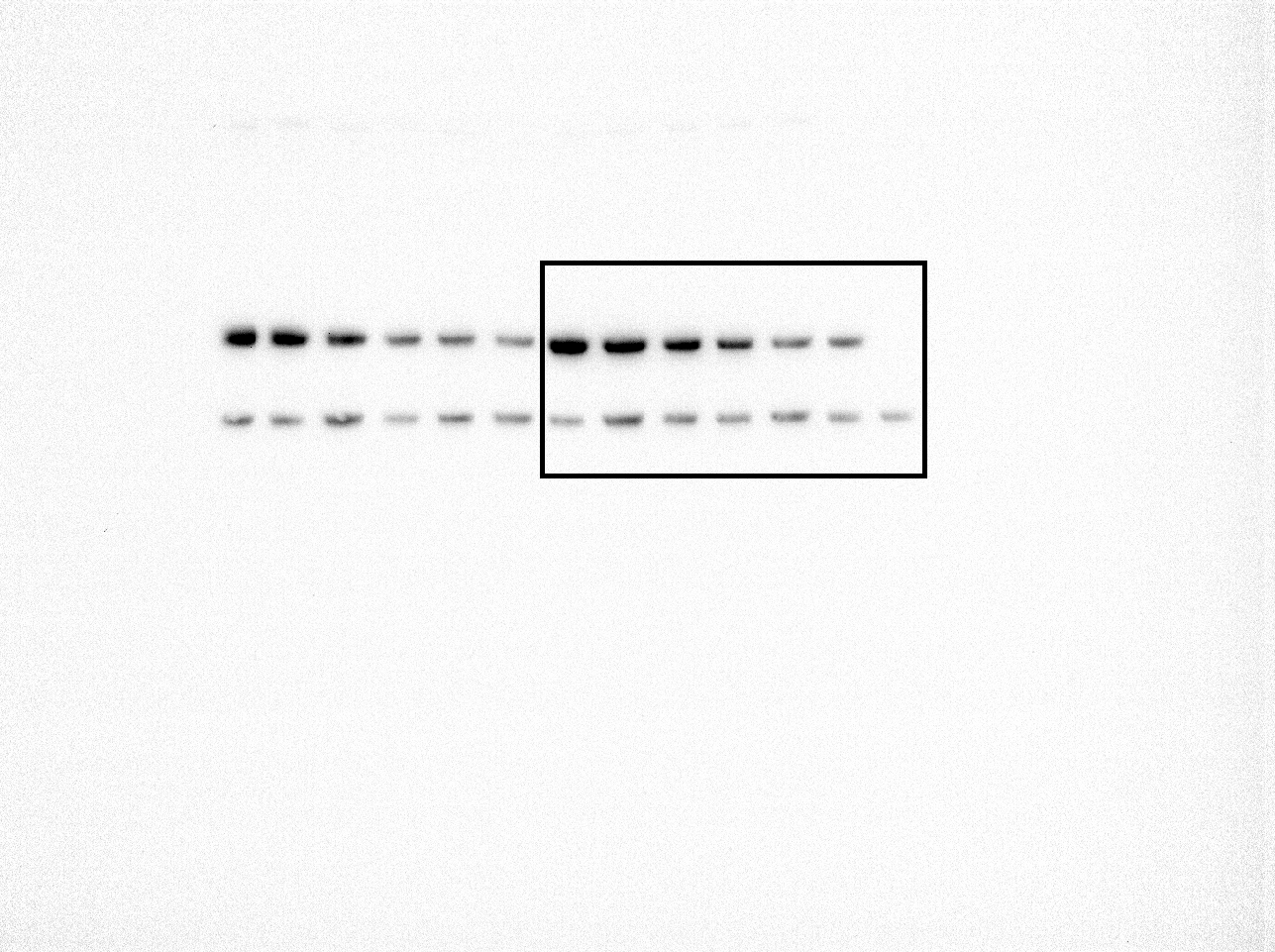

Supplement: Supplementary file 9 — Source Data for Expanded View [file EMBJ-41-e111608-s010.zip › SourceData/Figure_EV3/FEV3d/FEV3d_original_WB.tif]

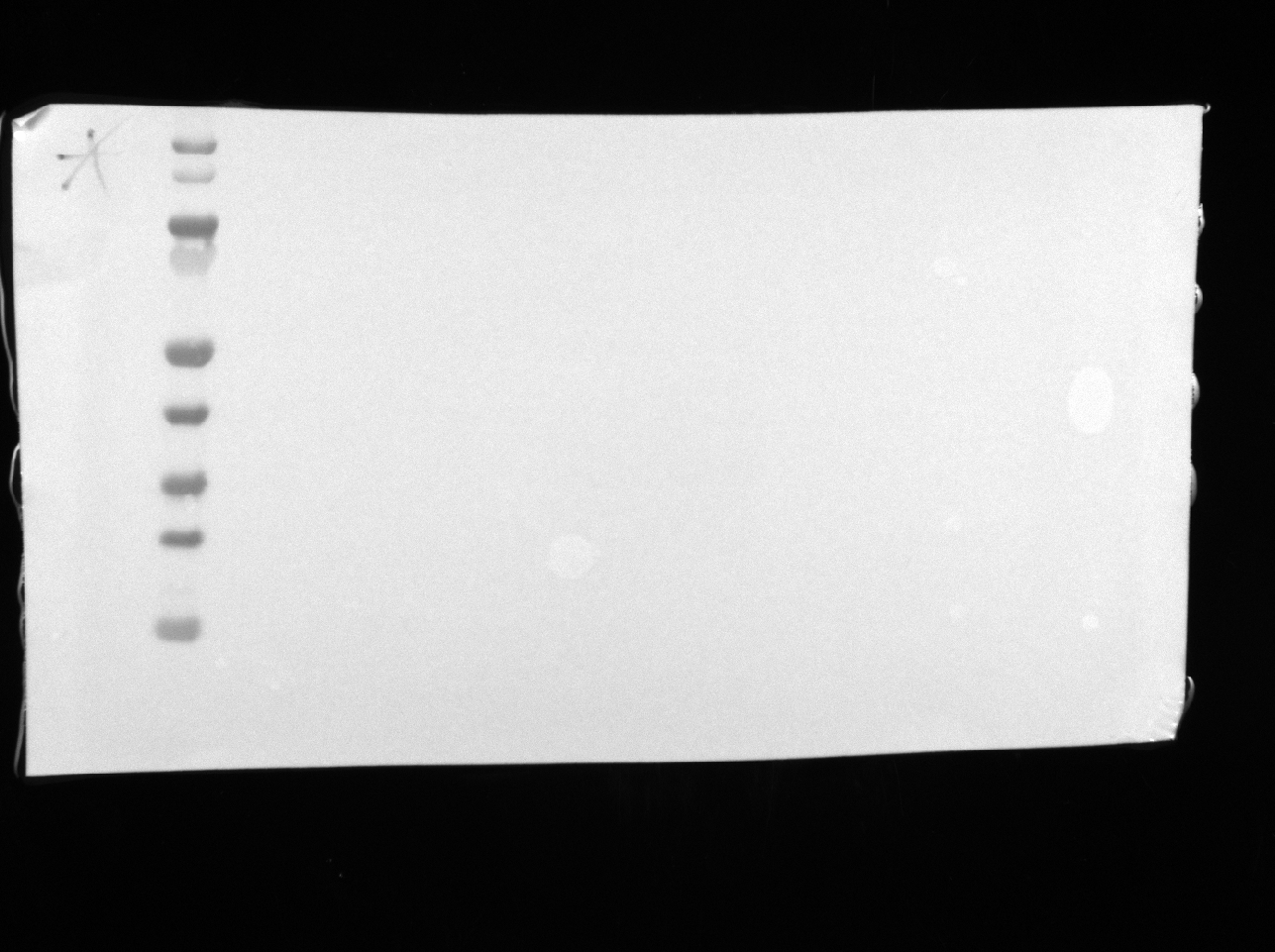

Supplement: Supplementary file 9 — Source Data for Expanded View [file EMBJ-41-e111608-s010.zip › SourceData/Figure_EV3/FEV3d/FEV3d_original_WB_marker.tif]

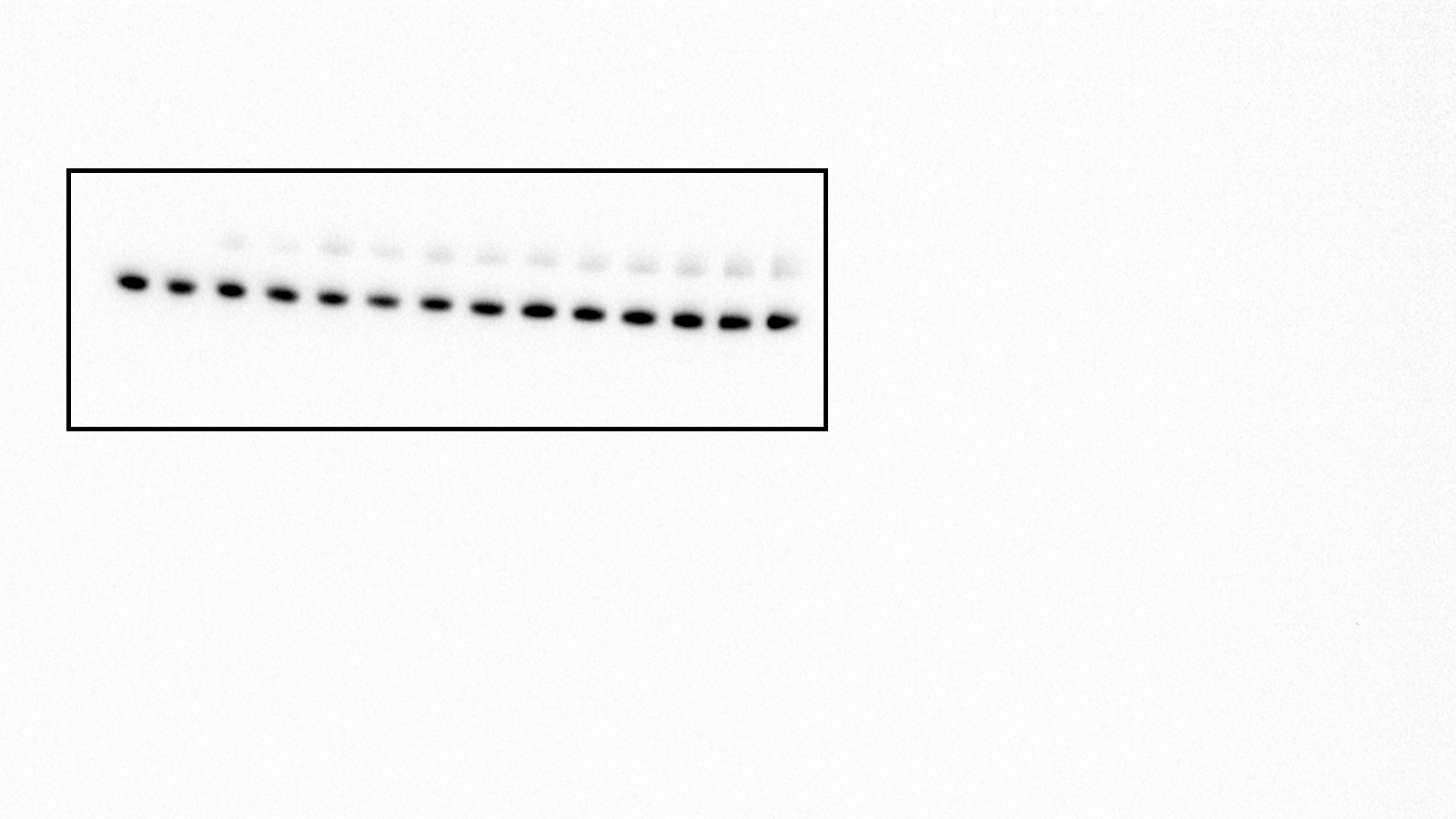

Supplement: Supplementary file 9 — Source Data for Expanded View [file EMBJ-41-e111608-s010.zip › SourceData/Figure_EV3/FEV3e/FEV3e_original_WB_ACTB.tif]

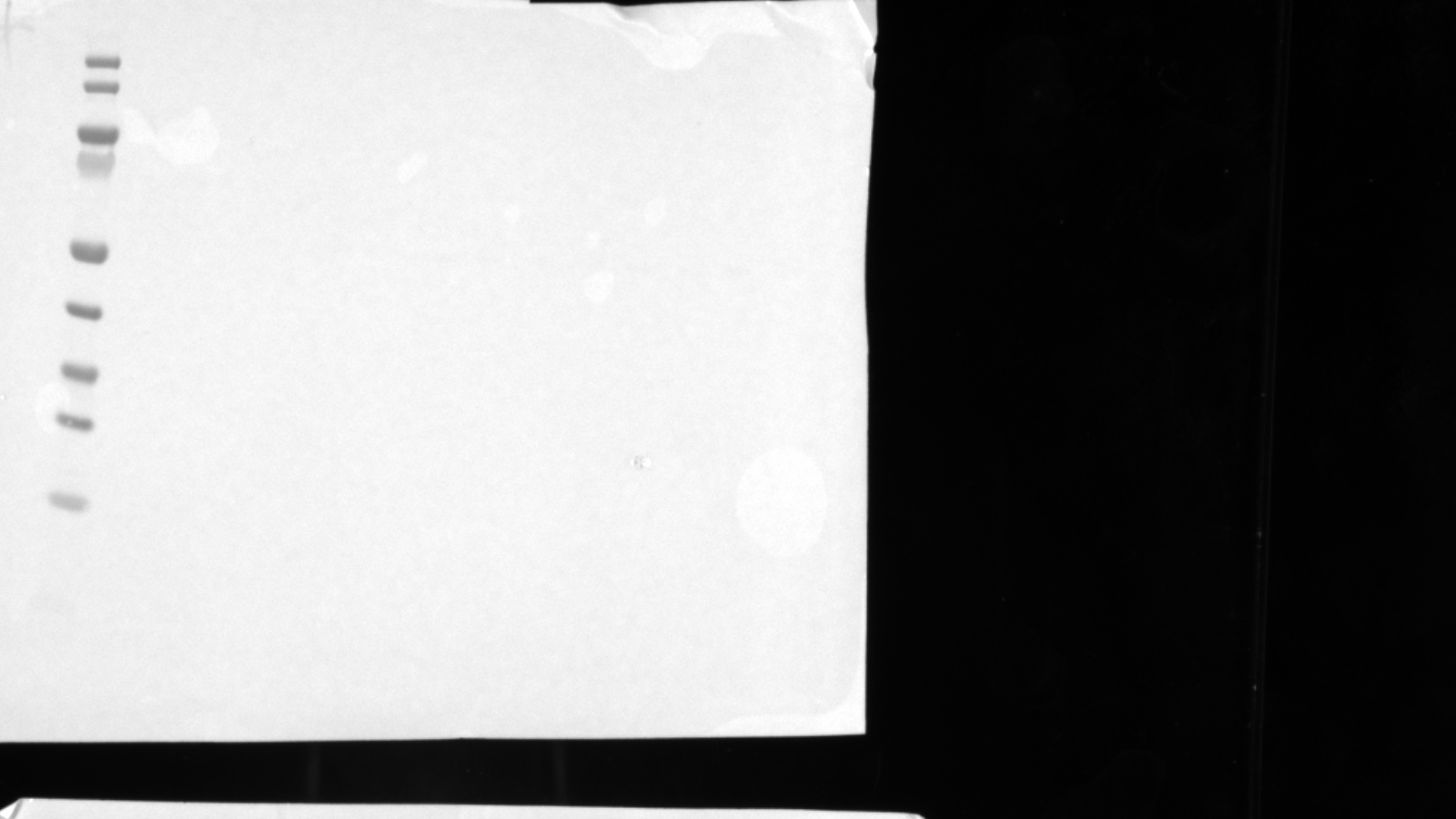

Supplement: Supplementary file 9 — Source Data for Expanded View [file EMBJ-41-e111608-s010.zip › SourceData/Figure_EV3/FEV3e/FEV3e_original_WB_ACTB_marker.tif]

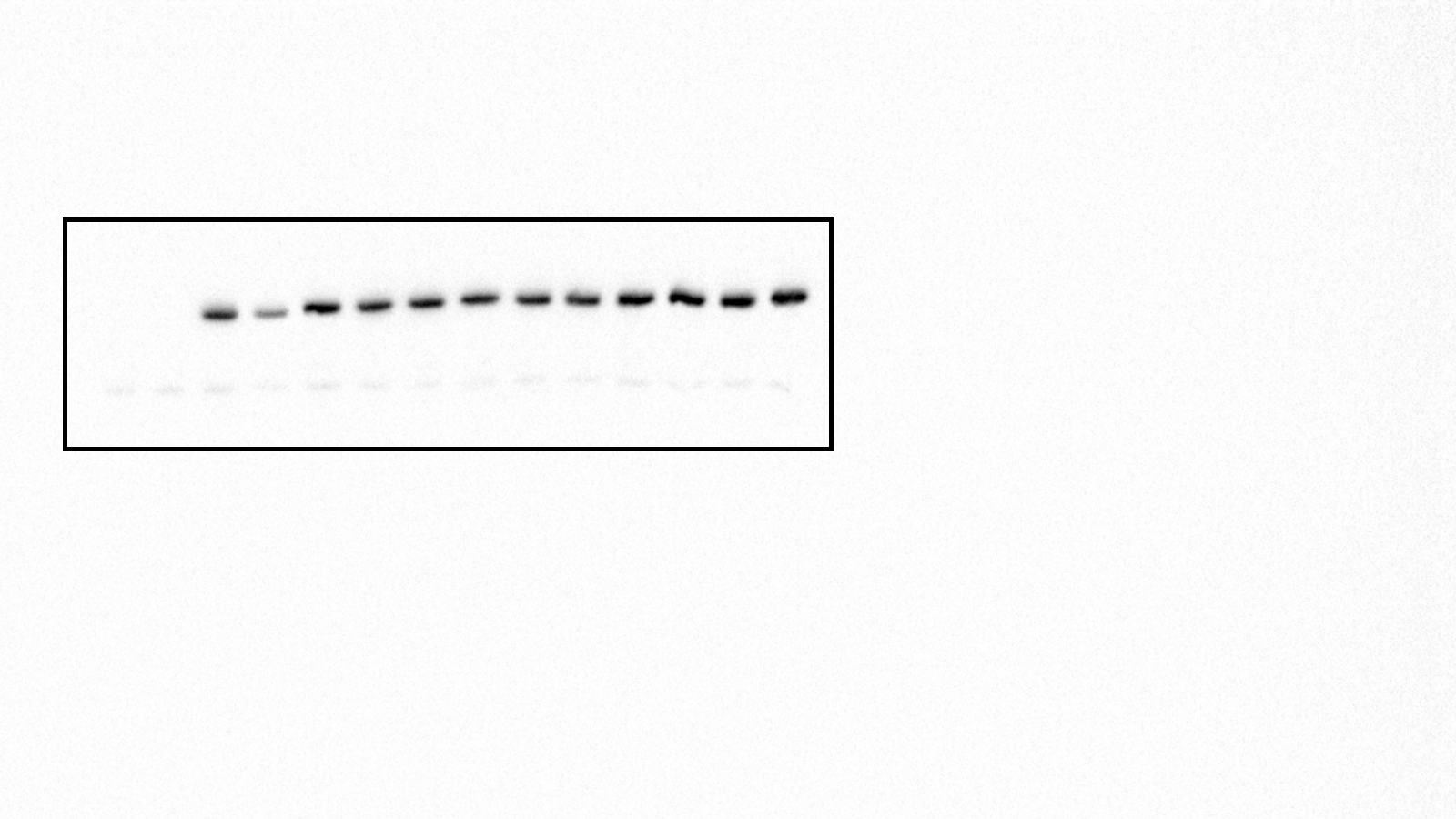

Supplement: Supplementary file 9 — Source Data for Expanded View [file EMBJ-41-e111608-s010.zip › SourceData/Figure_EV3/FEV3e/FEV3e_original_WB_NP.tif]

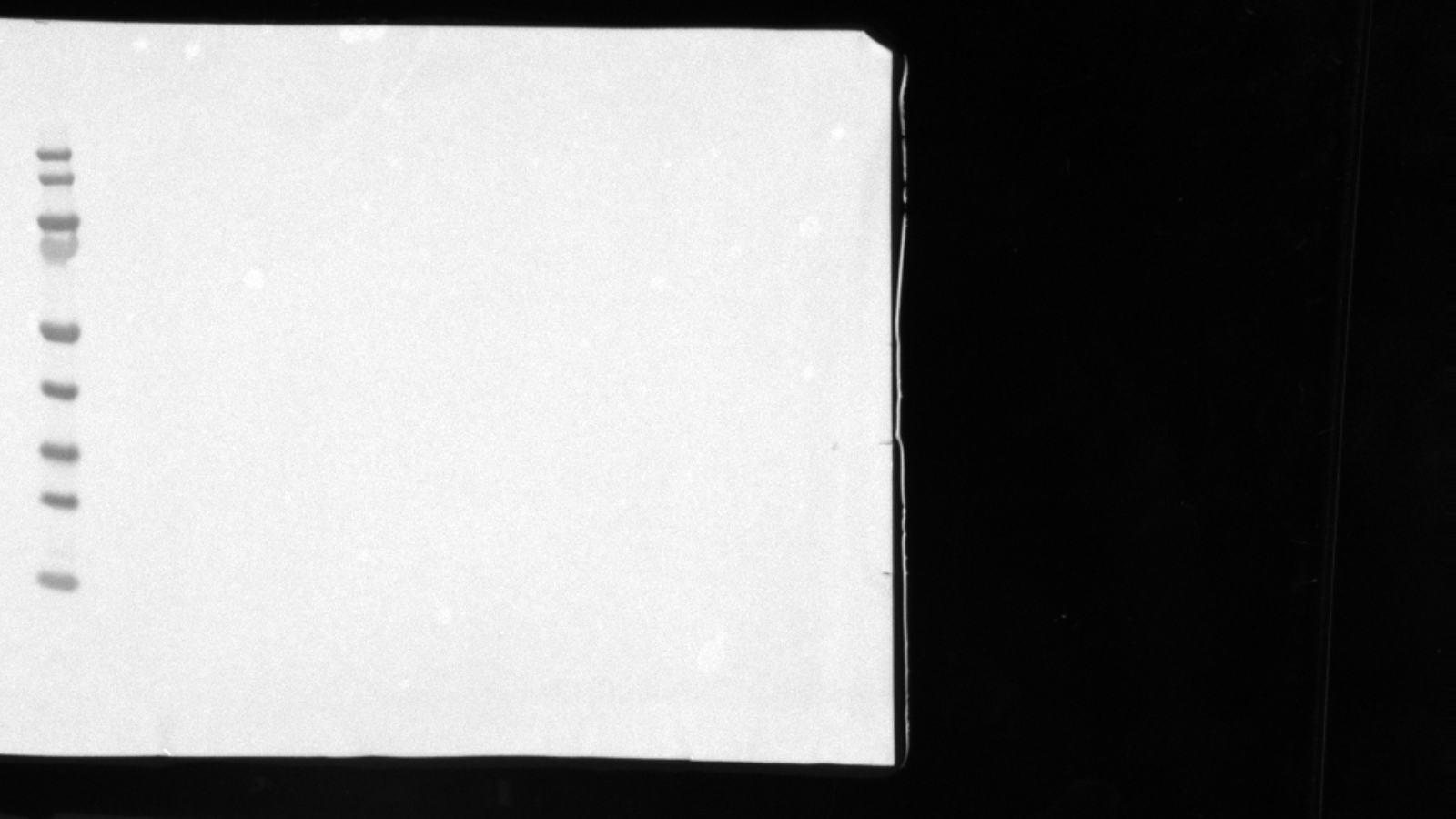

Supplement: Supplementary file 9 — Source Data for Expanded View [file EMBJ-41-e111608-s010.zip › SourceData/Figure_EV3/FEV3e/FEV3e_original_WB_NP_marker.tif]

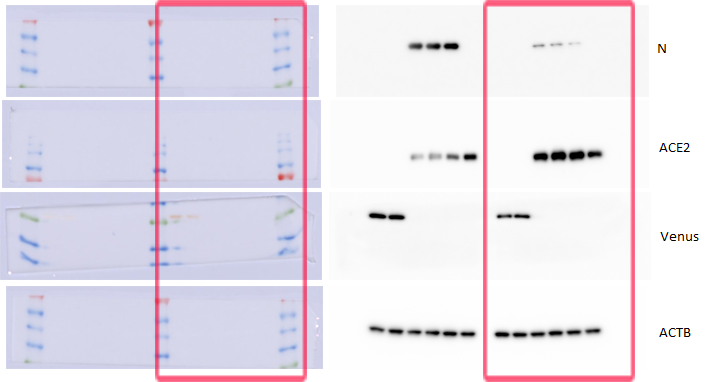

Supplement: Supplementary file 10 — Source Data for Figure 1 [file EMBJ-41-e111608-s004.png]
