# Supplementary material for: Attenuation of SARS‐CoV‐2 replication and associated inflammation by concomitant targeting of viral and host cap 2'‐O‐ribose methyltransferases
Source: EMBO J. 2022 Jul 25;41(17):e111608. doi: 10.15252/embj.2022111608 (PMC9350232; doi:10.15252/embj.2022111608)
Supplement: Supplementary file 26 [file EMBJ-41-e111608-s005.pdf]

THE EMBO JOURNAL

Attenuation of SARS-CoV-2 replication and associated inflammation by concomitant targeting of viral and host cap 2'-O-ribose methyltransferases

Valter Bergant, Shintaro Yamada, Vincent Grass, Yuta Tsukamoto, Teresa Lavacca, Karsten Krey, Maria-Teresa Mühlhofer, Sabine Wittmann, Armin Ensser, Alexandra Herrmann, Anja Hemdt, Yuriko Tomita, Shutoku Matsuyama, Takatsugu Hirokawa, Yiqi Huang, Antonio Piras, Constanze Jakwerth, Madlen Oelsner, Susanne Thieme, Alexander Graf, Stefan Krebs, Helmut Blum, Beate Kümmerer, Alexey Stukalov, Carsten Schmidt-Weber, Manabu IGARASHI, Thomas Gramberg, Andreas Pichlmair, and Hiroki Kato
DOI: 10.15252/embj.2022111608

Corresponding author(s): Andreas Pichlmair (andreas.pichlmair@tum.de) , Hiroki Kato (hkato@uni-bonn.de), Andreas Pichlmair (andreas.pichlmair@tum.de)

Review Timeline:

Submission Date:	8th May 22
Editorial Decision:	25th May 22
Revision Received:	13th Jun 22
Editorial Decision:	23rd Jun 22
Revision Received:	23rd Jun 22
Accepted:	27th Jun 22

Editor: Karin Dumstrei

Transaction Report:

(Please note that the manuscript was previously reviewed at another journal and the reports were taken into account in the decision making process at The EMBO Journal. Since the original reviews are not subject to EMBO's transparent review process policy, the reports and author response cannot be published

Note: With the exception of the correction of typographical or spelling errors that could be a source of ambiguity, letters and reports are not edited. Depending on transfer agreements, referee reports obtained elsewhere may or may not be included in this compilation. Referee reports are anonymous unless the Referee chooses to sign their reports.)

1st Editorial Decision 25th May 2022

Dear Andreas,

Thank you for submitting your manuscript to The EMBO Journal. This manuscript has been reviewed at another journal and transferred with referee reports and point-by-point response to The EMBO Journal.

I involved an arbitrating referee who looked at the manuscript and point-by-point response. As you can see below the referee finds the analysis insightful and important. The referee raises a few issues that would be good to sort out.

When you submit the revised version will you also please take care of the following issues:

- Upload a word doc of the text file

- We are missing 3-5 keywords

- We need a Author Contribution section

- Competing Interests should be called Disclosure and Competing Interests Statement.

- We need an author checklist - see also guide to authors

- We generally only allow 3 1st authors - it is not clear from the legend if you have 2 or 4.

- The reference list needs to be updated to EMBO Journal style.

- Regarding the figures: we no longer have supplemental figures, but instead have expanded view figures that are typeset and integrated into the html file. We allow only 5 expanded view figures and the rest would have to be added to an appendix. The appendix would also need a ToC. Alternatively, you can turn the supplementary figures into regular figures. See also our guide to authors or contact me if you need more help.

- Some tables are too big and will not convert to pdf. They should be changed to Datasets (this way they will stay as excel files) and called out as Dataset EV1 with legends in a separate tab within the xlsx sheet. See also our guide to authors or contact me if you need more help.

- Please double check that the funding is also entered into the online submission system =>Association's Initiative and Networking Fund (KA1-Co-02 "COVIPA"),BMK (A04) and HK (B22), TRR179 (TP11), PI 1084/4, PI 1084/5 and PI 1084/7 are missing from EJP system.

- We need a Data Availability section. This is the place to enter accession numbers etc. It should be place after the Materials and methods and before Acknowledgements. If no data needs to deposited in a database please add: This study includes no data deposited in external repositories.

- We include a synopsis of the paper that is visible on the html file (see http://emboj.embopress.org/). Can you provide me with a general summary statement and 3-5 bullet points that capture the key findings of the paper?

- It would also be good if you could send me a summary figure for the synopsis. The size should be 550 wide by 400 high (pixels).

That should be all! Let me know if you have any further questions

Best

Karin

Karin Dumstrei, PhD
Senior Editor
The EMBO Journal

Instructions for preparing your revised manuscript:

Guide For Authors: https://www.embopress.org/page/journal/14602075/authorguide

Use the link below to submit your revision:

https://emboj.msubmit.net/cgi-bin/main.plex

Referee #1:

I have now read the manuscript as well as the rebuttal letter co-submitted by the authors.

Overall I find this as a strong and important paper but of course it has lost a bit of its flashing novelty due to other recent published papers.
Nevertheless, I do endorse it for publication and I do not agree to the critics raised by reviewer 2.

The authors have addressed the comments by Reviewer 1 and 3 appropriately and I really enjoyed the flow the paper was written in and the overall conclusions.
This here is a school book example of how we can exploit the concept of repurposing drugs developed for other modalities and by using novel virological mechanistic understanding we can find drugs applicable to treat a new emerging disease.

I have some minor issues with data missing in the manuscript that should be addressed:

line 175 -> the MOI used is extremely low. Would be good to include a statement for why this approach was used -> or confirm data with a higher MOI
line 233 -> will be important to show ICE analysis and WB results for the proposed MTr1 KO cell line
line 272-274 -> will be important to show ICE analysis and WB results for the proposed KO cell lines

Figure 2 -> lack stats on panel c, d and e

Referee #1

I have now read the manuscript as well as the rebuttal letter co-submitted by the authors.

Overall I find this as a strong and important paper but of course it has lost a bit of its flashing novelty due to other recent published papers.

Nevertheless, I do endorse it for publication and I do not agree to the critics raised by reviewer 2.

The authors have addressed the comments by Reviewer 1 and 3 appropriately and I really enjoyed the flow the paper was written in and the overall conclusions.

This here is a school book example of how we can exploit the concept of repurposing drugs developed for other modalities and by using novel virological mechanistic understanding we can find drugs applicable to treat a new emerging disease.

We thank the reviewer for reviewing our work and recognizing the value in it.

I have some minor issues with data missing in the manuscript that should be addressed:

line 175 -> the MOI used is extremely low. Would be good to include a statement for why this approach was used -> or confirm data with a higher MOI

We thank the reviewer for this comment and recognize its importance. Many compounds exhibit best antiviral efficacy upon infection with low virus dose and show diminished efficacy upon increasing MOIs. We therefore chose to use low MOI infections for antiviral efficacy screenings. We demonstrate the antiviral efficacy of Tubercidin upon infections with wild-type SARS-CoV-2 employing MOI 0.1 (versus 0.01 as used in the experiment related to this comment) in Figure 1f, g. Figure 3f furthermore depicts results obtained from treating cells upon infection with SARS-CoV-2-GFP reporter virus at MOI 3.

line 233 -> will be important to show ICE analysis and WB results for the proposed MTr1 KO cell line

We thank the reviewer for this comment and provide WB analysis in Figure EV1j.

line 272-274 -> will be important to show ICE analysis and WB results for the proposed KO cell lines

We thank the reviewer for this comment and provide RT-qPCR analysis of gene expression in Figure EV2a.

Figure 2 -> lack stats on panel c, d and e

In Figure 2e, the high amount of missing values due to the detection limit of the plaque assay impedes statistical handling of the data. The biological difference is nevertheless clear, and the lack of statistics does not impair the interpretation of the panels. For consistency reasons, we left out the statistics also in Figure 2d.

In Figure 2c, we could not observe any biologically meaningful trends in the data, even though some differences may be statistically significant (due to low data scattering, etc.). Adding statistics to this panel may be misleading to the readers, if misinterpreted as something biologically relevant. For this reason, we ask the reviewer for understanding to leave out the statistical analysis for this particular panels.

Hi Andreas,

Thanks for sending me the revised version. I have looked at it and all looks good. I am therefore pleased to let you know that I will accept the manuscript for publication here. Before sending you the formal acceptance letter there are just a few formatting issues that we need to sort out:

Reference list: Please reduce the number of authors in the Perez-Riverol et reference to 10 authors et al.

We don't allow Data Not Shown (page 8) - please rephrase

We are missing the ORCID ID for Kato

Please include the funding information in the Acknowledgements.

The legends for the dataset EV tables need to be removed from the manuscript file and added as a separate tab in the excel files.

The Appendix needs a ToC and the figure legend removed from the MS and added to the Appendix file.

Please double check that the MS sections are in the right order

Correct "Summary" needs correcting to 'Abstract'.

Correct "Figure text" to 'Figure Legends' and "Expanded View Figure Text" to 'Expanded View Figure Legends'.

Please make sure to make the dataset PXD034361 is made public

While checking the SD I have noted that for Figure EV1 that the the second raw of images in the figure panel FEV1h is missing (the 3 images A549 + IFN-alpha). I am just pointing this out in case it was overlooked.

Our publisher has also done their pre-publication check on your manuscript. When you log into the manuscript submission system you will see the file "Data Edited Manuscript file". Please take a look at the word file and the comments regarding the figure legends and respond to the issues.

Please upload a clean MS version and a point-by-point response to the editorial points. You can remove synopsis text from the MS as it is uploaded as a separate file.

Once I get the revised version in then I will move forward with the acceptance of the MS for publication here.

With best wishes

Karin

Karin Dumstrei, PhD
Senior Editor
The EMBO Journal

link to submit your revision:

https://emboj.msubmit.net/cgi-bin/main.plex

Thanks for sending me the revised version. I have looked at it and all looks good. I am therefore pleased to let you know that I will accept the manuscript for publication here. Before sending you the formal acceptance letter there are just a few formatting issues that we need to sort out:

Reference list: Please reduce the number of authors in the Perez-Riverol et reference to 10 authors et al.

Done.

We don't allow Data Not Shown (page 8) - please rephrase

The phrase was removed.

We are missing the ORCID ID for Kato

Done.

Please include the funding information in the Acknowledgements.

Done.

The legends for the dataset EV tables need to be removed from the manuscript file and added as a separate tab in the excel files.

Done.

The Appendix needs a ToC and the figure legend removed from the MS and added to the Appendix file.

Appendix table of content added as a separate file.

Please double check that the MS sections are in the right order

Done. Some sections were moved to follow section order as specified in author guideliness.

Correct "Summary" needs correcting to 'Abstract'.

Done.

Correct "Figure text" to 'Figure Legends' and "Expanded View Figure Text" to 'Expanded View Figure Legends'.

Done.

Please make sure to make the dataset PXD034361 is made public

We submitted the request to PRIDE – the dataset should become accessible in the next few days.

While checking the SD I have noted that for Figure EV1 that the the second raw of images in the figure panel FEV1h is missing (the 3 images A549 + IFN-alpha). I am just pointing this out in case it was overlooked.

We apologise, this was overlooked. Was added.

Our publisher has also done their pre-publication check on your manuscript. When you log into the manuscript submission system you will see the file "Data Edited Manuscript file". Please take a look at the word file and the comments regarding the figure legends and respond to the issues.

Done.

Please upload a clean MS version and a point-by-point response to the editorial points. You can remove synopsis text from the MS as it is uploaded as a separate file.

Done.

Dear Andreas and Hiroki,

Thank you for submitting the revised version to The EMBO Journal. I have now had a chance to take a look at it and all looks good.

I am therefore very pleased to accept the MS for publication in The EMBO Journal.

Congratulations on a nice study!

With best wishes

Karin

Karin Dumstrei, PhD
Senior Editor
The EMBO Journal

Please note that it is EMBO Journal policy for the transcript of the editorial process (containing referee reports and your response letter) to be published as an online supplement to each paper. If you do NOT want this, you will need to inform the Editorial Office via email immediately. More information is available here:
https://www.embopress.org/page/journal/14602075/authorguide#transparentprocess

Your manuscript will be processed for publication in the journal by EMBO Press. Manuscripts in the PDF and electronic editions of The EMBO Journal will be copy edited, and you will be provided with page proofs prior to publication. Please note that supplementary information is not included in the proofs.

You will be contacted by Wiley Author Services to complete licensing and payment information. The required 'Page Charges Authorization Form' is available here: https://www.embopress.org/pb-assets/embo-site/tej_apc.pdf - please download and complete the form and return to embopressproduction@wiley.com

Should you be planning a Press Release on your article, please get in contact with embojournal@wiley.com as early as possible, in order to coordinate publication and release dates.

If you have any questions, please do not hesitate to call or email the Editorial Office. Thank you for your contribution to The EMBO Journal.

** Click here to be directed to your login page: https://emboj.msubmit.net

EMBO Press Author Checklist

Corresponding Author Name: Andreas Pichlmair and Hiroki Kato
Journal Submitted to: EMBO
Manuscript Number: EMBOJ-2022- 111608

USEFUL LINKS FOR COMPLETING THIS FORM
The EMBO Journal - Author Guidelines
EMBO Reports - Author Guidelines
Molecular Systems Biology - Author Guidelines
EMBO Molecular Medicine - Author Guidelines

Reporting Checklist for Life Science Articles (updated January 2022)
This checklist is adapted from Materials Design Analysis Reporting (MDAR) Checklist for Authors. MDAR establishes a minimum set of requirements in transparent reporting in the life sciences (see Statement of Task: 10.31222/osf.io/9sm4x). Please follow the journal's guidelines in preparing your manuscript.
Please note that a copy of this checklist will be published alongside your article.

Abridged guidelines for figures
1. Data
The data shown in figures should satisfy the following conditions:

- the data were obtained and processed according to the field's best practice and are presented to reflect the results of the experiments in an accurate and unbiased manner.
- ideally, figure panels should include only measurements that are directly comparable to each other and obtained with the same assay.
- plots include clearly labeled error bars for independent experiments and sample sizes. Unless justified, error bars should not be shown for technical replicates.
- if n<5, the individual data points from each experiment should be plotted. Any statistical test employed should be justified.
- Source Data should be included to report the data underlying figures according to the guidelines set out in the authorship guidelines on Data Presentation.

2. Captions
Each figure caption should contain the following information, for each panel where they are relevant:

- a specification of the experimental system investigated (eg cell line, species name).
- the assay(s) and method(s) used to carry out the reported observations and measurements.
- an explicit mention of the biological and chemical entity(ies) that are being measured.
- an explicit mention of the biological and chemical entity(ies) that are altered/varied/perturbed in a controlled manner.
- the exact sample size (n) for each experimental group/condition, given as a number, not a range;
- a description of the sample collection allowing the reader to understand whether the samples represent technical or biological replicates (including how many animals, litters, cultures, etc.).
- a statement of how many times the experiment shown was independently replicated in the laboratory.
- definitions of statistical methods and measures:
  - common tests, such as t-test (please specify whether paired vs. unpaired), simple χ2 tests, Wilcoxon and Mann-Whitney tests, can be unambiguously identified by name only, but more complex techniques should be described in the methods section;
  - are tests one-sided or two-sided?
  - are there adjustments for multiple comparisons?
  - exact statistical test results, e.g., P values = x but not P values < x;
  - definition of 'center values' as median or average;
  - definition of error bars as s.d. or s.e.m.

Please complete ALL of the questions below.
Select "Not Applicable" only when the requested information is not relevant for your study.

Materials

Newly Created Materials	Information included in the manuscript?	In which section is the information available? (Reagents and Tools Table, Materials and Methods, Figures, Data Availability Section)
New materials and reagents need to be available; do any restrictions apply?	Not Applicable	

Antibodies	Information included in the manuscript?	In which section is the information available? (Reagents and Tools Table, Materials and Methods, Figures, Data Availability Section)
For **antibodies** provide the following information:<br>- Commercial antibodies: RRID (if possible) or supplier name, catalogue number and or/clone number<br>- Non-commercial: RRID or citation	Yes	Materials and Methods

DNA and RNA sequences	Information included in the manuscript?	In which section is the information available? (Reagents and Tools Table, Materials and Methods, Figures, Data Availability Section)
**Short novel DNA or RNA including primers, probes**: provide the sequences.	Yes	Materials and Methods

Cell materials	Information included in the manuscript?	In which section is the information available? (Reagents and Tools Table, Materials and Methods, Figures, Data Availability Section)
**Cell lines:** Provide species information, strain. Provide accession number in repository **OR** supplier name, catalog number, clone number, and/**OR** RRID.	Yes	Materials and Methods
**Primary cultures:** Provide species, strain, sex of origin, genetic modification status.	Yes	Materials and Methods
Report if the cell lines were recently **authenticated** (e.g., by STR profiling) and tested for mycoplasma contamination.	Yes	Materials and Methods

Experimental animals	Information included in the manuscript?	In which section is the information available? (Reagents and Tools Table, Materials and Methods, Figures, Data Availability Section)
**Laboratory animals or Model organisms:** Provide species, strain, sex, age, genetic modification status. Provide accession number in repository **OR** supplier name, catalog number, clone number, **OR** RRID.	Yes	Materials and Methods
**Animal observed in or captured from the field:** Provide species, sex, and age where possible.	Not Applicable	
Please detail **housing and husbandry conditions**.	Yes	Materials and Methods

Plants and microbes	Information included in the manuscript?	In which section is the information available? (Reagents and Tools Table, Materials and Methods, Figures, Data Availability Section)
**Plants:** provide species and strain, ecotype and cultivar where relevant, unique accession number if available, and source (including location for collected wild specimens).	Not Applicable	
**Microbes:** provide species and strain, unique accession number if available, and source.	Yes	Materials and Methods

Human research participants	Information included in the manuscript?	In which section is the information available? (Reagents and Tools Table, Materials and Methods, Figures, Data Availability Section)
If collected and within the bounds of privacy constraints report on age, sex and gender or ethnicity for all study participants.	Not Applicable	

Core facilities	Information included in the manuscript?	In which section is the information available? (Reagents and Tools Table, Materials and Methods, Figures, Data Availability Section)
If your work benefited from core facilities, was their service mentioned in the acknowledgments section?	Yes	Acknowledgements

Design

Study protocol	Information included in the manuscript?	In which section is the information available? (Reagents and Tools Table, Materials and Methods, Figures, Data Availability Section)
If study protocol has been **pre-registered, provide DOI in the manuscript**. For clinical trials, provide the trial registration number **OR** cite DOI.	Not Applicable	
Report the **clinical trial registration number** (at ClinicalTrials.gov or equivalent), where applicable.	Not Applicable	

Laboratory protocol	Information included in the manuscript?	In which section is the information available? (Reagents and Tools Table, Materials and Methods, Figures, Data Availability Section)
Provide DOI OR other citation details if **external detailed step-by-step protocols** are available.	Yes	Materials and Methods

Experimental study design and statistics	Information included in the manuscript?	In which section is the information available? (Reagents and Tools Table, Materials and Methods, Figures, Data Availability Section)
Include a statement about **sample size** estimate even if no statistical methods were used.	Yes	Figure legends, Materials and Methods
Were any steps taken to minimize the effects of subjective bias when allocating animals/samples to treatment (e.g. **randomization procedure**)? If yes, have they been described?	Not Applicable	
Include a statement about **blinding** even if no blinding was done.	Not Applicable	No blind handling was performed.
Describe **inclusion/exclusion criteria** if samples or animals were excluded from the analysis. Were the criteria pre-established?<br><br>If sample or data points were omitted from analysis, report if this was due to attrition or intentional exclusion and provide justification.	Yes	Materials and Methods
For every figure, are **statistical tests** justified as appropriate? Do the data meet the assumptions of the tests (e.g., normal distribution)? Describe any methods used to assess it. Is there an estimate of variation within each group of data? Is the variance similar between the groups that are being statistically compared?	Yes	Figure legends, Materials and Methods

Sample definition and in-laboratory replication	Information included in the manuscript?	In which section is the information available? (Reagents and Tools Table, Materials and Methods, Figures, Data Availability Section)
In the figure legends: state number of times the experiment was **replicated** in laboratory.	Yes	Figure legends
In the figure legends: define whether data describe **technical or biological replicates**.	Yes	Figure legends

Ethics

Ethics	Information included in the manuscript?	In which section is the information available? (Reagents and Tools Table, Materials and Methods, Figures, Data Availability Section)
Studies involving **human participants**: State details of **authority granting ethics approval** (IRB or equivalent committee(s), provide reference number for approval.	Not Applicable	
Studies involving **human participants**: Include a statement confirming that **informed consent** was obtained from all subjects and that the experiments conformed to the principles set out in the WMA Declaration of Helsinki and the Department of Health and Human Services Belmont Report.	Not Applicable	
Studies involving **human participants:** For publication of **patient photos**, include a statement confirming that consent to publish was obtained.	Not Applicable	
Studies involving experimental **animals**: State details of **authority granting ethics approval** (IRB or equivalent committee(s), provide reference number for approval. Include a statement of compliance with ethical regulations.	Yes	Materials and Methods
Studies involving **specimen and field samples:** State if relevant **permits** obtained, provide details of authority approving study; if none were required, explain why.	Not Applicable	

Dual Use Research of Concern (DURC)	Information included in the manuscript?	In which section is the information available? (Reagents and Tools Table, Materials and Methods, Figures, Data Availability Section)
Could your study fall under dual use research restrictions? Please check biosecurity documents and list of **select agents and toxins** (CDC): https://www.selectagents.gov/sat/list.htm	Not Applicable	
If you used a select agent, is the security level of the lab appropriate and reported in the manuscript?	Not Applicable	
If a study is subject to dual use research of concern regulations, is the name of the **authority granting approval and reference number** for the regulatory approval provided in the manuscript?	Not Applicable	

Reporting

The MDAR framework recommends adoption of discipline-specific guidelines, established and endorsed through community initiatives. Journals have their own policy about requiring specific guidelines and recommendations to complement MDAR.

Adherence to community standards	Information included in the manuscript?	In which section is the information available? (Reagents and Tools Table, Materials and Methods, Figures, Data Availability Section)
State if relevant guidelines or checklists (e.g., **ICMJE, MIBBI, ARRIVE, PRISMA**) have been followed or provided.	Not Applicable	
For **tumor marker prognostic studies,** we recommend that you follow the **REMARK** reporting guidelines (see link list at top right). See author guidelines, under 'Reporting Guidelines'. Please confirm you have followed these guidelines.	Not Applicable	
For **phase II and III randomized controlled trials**, please refer to the **CONSORT** flow diagram (see link list at top right) and submit the CONSORT checklist (see link list at top right) with your submission. See author guidelines, under 'Reporting Guidelines'. Please confirm you have submitted this list.	Not Applicable	

Data Availability

Data availability	Information included in the manuscript?	In which section is the information available? (Reagents and Tools Table, Materials and Methods, Figures, Data Availability Section)
Have **primary datasets** been deposited according to the journal's guidelines (see 'Data Deposition' section) and the respective accession numbers provided in the Data Availability Section?	Yes	Materials and Methods
Were **human clinical and genomic datasets** deposited in a public access-controlled repository in accordance to ethical obligations to the patients and to the applicable consent agreement?	Not Applicable	
Are **computational models** that are central and integral to a study available without restrictions in a machine-readable form? Were the relevant accession numbers or links provided?	Not Applicable	
If publicly available data were reused, provide the respective **data citations in the reference list**.	Not Applicable	
